# Overexpressing the N‐terminus of *CATALASE2* enhances plant jasmonic acid biosynthesis and resistance to necrotrophic pathogen *Botrytis cinerea* B05.10

**DOI:** 10.1111/mpp.13106

**Published:** 2021-07-10

**Authors:** Yu Zhang, Ru‐Feng Song, Hong‐Mei Yuan, Ting‐Ting Li, Lin‐Feng Wang, Kai‐Kai Lu, Jia‐Xing Guo, Wen‐Cheng Liu

**Affiliations:** ^1^ State Key Laboratory of Crop Stress Adaptation and Improvement School of Life Sciences Henan University Kaifeng China; ^2^ Hainan Key Laboratory for Sustainable Utilization of Tropical Bioresources College of Tropical Crops Hainan University Haikou China; ^3^ Jiangsu Key Laboratory of Marine Pharmaceutical Compound Screening Jiangsu Ocean University Lianyungang China

**Keywords:** acyl‐CoA oxidases, *Botrytis cinerea*, catalase 2, jasmonic acid, salicylic acid

## Abstract

Salicylic acid (SA) acts antagonistically to jasmonic acid (JA) in plant immunity. We previously reported that CATALASE2 (CAT2) promotes JA‐biosynthetic acyl‐CoA oxidase (ACX) activity to enhance plant resistance to necrotrophic *Botrytis cinerea*, and SA represses JA biosynthesis through inhibiting CAT2 activity, while the underlying mechanism remains to be further elucidated. Here, we report that the truncated CAT2 N‐terminus (CAT2‐N) interacts with and promotes ACX2/3, and *CAT2‐N*‐overexpressing plants have increased JA accumulation and enhanced resistance to *B*. *cinerea* B05.10, but compromised antagonism of SA on JA. Catalase inhibitor treatment or mutating CAT2 active amino acids abolished CAT2 H_2_O_2_‐decomposing activity but did not affect its promotion of ACX2/3 activity via interaction. CAT2‐N, a truncated protein with no catalase activity, interacted with and promoted ACX2/3. Overexpressing *CAT2‐N* in *Arabidopsis* plants resulted in increased ACX activity, higher JA accumulation, and stronger resistance to *B*. *cinerea* B05.10 infection. Additionally, SA dramatically repressed JA biosynthesis and resistance to *B*. *cinerea* in the wild type but not in the *CAT2‐N*‐overexpressing plants. Together, our study reveals that CAT2‐N can be utilized as an accelerator for JA biosynthesis during plant resistance to *B*. *cinerea* B05.10, and this truncated protein partly relieves SA repression of JA biosynthesis in plant defence responses.

## Graphical abstract

N‐terminal fragment of CATALASE2 interacts with and promotes acyl‐CoA oxidases implicated in JA biosynthesis and enhances plant resistance to *Botrytis* *cinerea*, thus CATALASE2‐N could be a genetic tool conferring plant resistance.

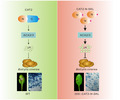

## 1 INTRODUCTION

Due to a sessile lifestyle, plants are constantly confronted with various biotic stresses, including fungal infection, bacterial invasion, and virus attack (Chaouch et al., 2010; Liu et al., 2020a; Qi et al., 2012). During the long‐term evolution of the arms race with pathogens, plants have evolved sophisticated defence mechanisms to effectively ward off different kinds of pathogens (Li et al., 2020b; Song et al., 2021; Yuan et al., 2017). Based on lifestyles, plant pathogens can be generally categorized into biotrophic and necrotrophic pathogens, which derive nutrients mainly from living host tissues or dead/dying cells, respectively (Qi et al., 2012; Spoel & Dong, 2008). Therefore, accurate activation of a defence strategy is the prerequisite for successful plant resistance in response to distinct pathogens with different lifestyles (Adedeji & Babalola, 2020; Ochoa‐Lopez et al., 2020; Zhang et al., 2020b).

Phytohormones play essential roles in plant resistance against pathogen infection through regulating the expression of a battery of genes related to pathogen recognition and defence response (Koornneef & Pieterse, 2008; Lopez et al., 2008). It has been widely documented that salicylic acid (SA) plays an essential role in plant resistance to biotrophic and semibiotrophic pathogens such as the model pathogen *Pseudomonas syringae* pv. *tomato* DC3000 (Pst DC3000) (Chen et al., 2020a; Peng et al., 2021; Zhang et al., 2020c). On infection with Pst DC3000, SA accumulation is rapidly induced in plants by the activation of SA biosynthesis and repression of SA metabolism, resulting in translocation of NONEXPRESSOR OF PATHOGENESIS‐RELATED GENES 1 (NPR1) from cytoplasm to nucleus. NPR1, as a master transcription regulator in the plant immune response, interacts with other transcription factors in the nucleus and modulates the expression of a large number of defence‐related genes, including multiple *pathogenesis‐related* (*PR*) genes (Budimir et al., 2021; Liu et al., 2020d; Spoel & Dong, 2008; Zavaliev et al., 2020). In addition, SA can also directly bind to and inhibit catalase (CAT), a key enzyme catalysing the degradation of H_2_O_2_, thus increasing H_2_O_2_ accumulation in plant cells (Chen et al., 1993b; Sanchez‐Casas & Klessig, 1994). High levels of H_2_O_2_ enhance plant pathogen resistance through directly killing the invasive pathogens as an oxidative antimicrobial chemical, strengthening the cell wall barrier as a cross‐linker for structural proteins, and also upregulating the expression of some defence‐related genes as a signalling molecule, thus serving as an important factor involved in SA‐mediated plant resistance to biotrophic pathogens (Bradley et al., 1992; Chen et al., 2020b; Jasso‐Robles et al., 2020; Shen et al., 2021; Su et al., 2018a; Zhao et al., 2021). Previous reports documented a vital role of CAT in the plant immune response. For example, repression of *CAT* expression in tobacco or mutation of *CAT2* in *Arabidopsis* results in enhanced resistance to bacterial pathogens with decreased catalase activity and increased H_2_O_2_ accumulation, while overexpressing *CAT1* dampens pathogen‐induced H_2_O_2_ accumulation and the defence response in tobacco (Chaouch et al., 2010; Mittler et al., 1999; Yuan et al., 2017).

*Botrytis cinerea* is a typical fungal pathogen with a necrotrophic lifestyle attacking over 200 crop hosts worldwide, which kills plant cells and consumes the nutrient contents (Shu et al., 2020; Williamson et al., 2007; Xiong et al., 2019). When infected by *B*. *cinerea*, plants activate both jasmonic acid (JA) biosynthesis and JA signalling to enhance their immune responses (Liao et al., 2020; Xiong et al., 2019). Lipids derived from membrane are catalysed as (9S,13S)‐*cis*‐(+)‐oxophytodienoic acid (OPDA) or dinor‐12‐oxo‐fitodienoic acid (dnOPDA) in plastids, and then imported and converted to 3‐oxo‐2‐(2′‐[Z]‐pentenyl)‐cyclopentane‐1‐octanoic acid‐coenzyme A (OPC8‐CoA) in peroxisomes (Liu et al., 2020c; Wang et al., 2020b; Zhang et al., 2020a). The OPC8‐CoA in peroxisomes is metabolized to OPC6‐CoA, subsequently OPC4‐CoA, and finally JA by three rounds of β‐oxidation cycle by a set of four enzymatic reactions: acyl‐CoA oxidase (ACX)‐mediated oxidation, multifunctional protein (MFP)‐mediated hydration and oxidation, and 3‐ketoacyl‐CoA‐thiolase (KAT)‐mediated thiolysis (Graham & Eastmond, 2002; Kelly et al., 2011; Wang et al., 2021; Yuan et al., 2017). *B. cinerea* infection‐induced JA binds to its receptor CORONATINE INSENSITIVE1 (COI1) and promotes the degradation of JAZ proteins, releasing the repression of downstream transcription factors such as MYC2, ERF1, and ORA59, and thus regulating the expression of downstream JA‐responsive genes (Aerts et al., 2021; Su et al., 2018b; Wang et al., 2020a; Zhang et al., 2019).

Although it is reported that SA elicits an antagonistic effect on JA in plant immune responses (Ndamukong et al., 2007; Spoel et al., 2003), SA also plays a role in plant resistance to *B. cinerea*. For example, repressing SA accumulation in *Arabidopsis* plants, by expressing bacterial *nahG* that encodes an SA hydroxylase or by applying a phenylalanine ammonia‐lyase inhibitor, significantly increases susceptibility to both Pst DC3000 and *B. cinerea* infection (Ferrari et al., 2003), revealing that both SA and JA are crucial phytohormones mediating plant defence responses to biotrophic and necrotrophic pathogens.

We previously reported that the *acx2‐1 acx3‐6* mutant with mutation of both *ACX2* and *ACX3* has extremely low acyl‐CoA oxidase activity with OPC4‐CoA as the substrate; thus, the mutant exhibited increased susceptibility to *B*. *cinerea* due to a decreased JA level, revealing a key role of ACX2/3 in *B. cinerea* infection‐induced JA biosynthesis (Yuan et al., 2017). We also found that CAT2 interacts with ACX2/3 in peroxisomes and promotes their activity to enhance JA biosynthesis, increasing plant resistance to necrotrophic *B*. *cinerea* B05.10 infection (Liu et al., 2017; Yuan et al., 2017). The *Arabidopsis cat2‐1* mutant has decreased ACX activity, repressed JA accumulation, and thus reduced resistance to *B*. *cinerea* B05.10 (Yuan et al., 2017). However, the mechanism underlying CAT2‐mediated promotion of ACX activity via their interaction remains elusive. Elucidating how CAT2 increases ACX activity would provide more insight into mechanism of the plant defence response.

Here, we report that the N‐terminus, but not the C‐terminus, of CAT2 interacts with and promotes ACX2/3, and overexpressing the *CAT2‐N* terminus enhances plant ACX activity, JA biosynthesis, and thus resistance to *B*. *cinerea* B05.10 infection. Our study showed that treatment of catalase inhibitor 3‐amino‐1,2,4‐triazole (3‐AT) or mutating CAT2 active amino acid sites necessary for catalase activity severely abolished CAT2 H_2_O_2_‐decomposing activity but did not affect CAT2 interaction with ACX2/3 and promotion on their ACX activity. The truncated N‐terminus of CAT2 (CAT2‐N) had no catalase activity but can also interact with and promote ACX2/3. Furthermore, we overexpressed *CAT2‐N* fused with type I peroxisomal targeting signal (PTS1) in wildtype *Arabidopsis* plants, and found that the transgenic 35S::*CAT2‐N‐PTS1* plants had increased ACX activity, JA accumulation, and showed enhanced resistance to *B*. *cinerea* B05.10 infection. In addition, the transgenic plants showed diminished antagonism of SA on JA biosynthesis. Together, our study reveals that CAT2‐N interacts with and promotes ACX2/3 implicated in JA biosynthesis and enhances plant resistance to *B*. *cinerea*, and this truncated protein partly relieves SA repression of JA biosynthesis in plant defence responses.

## 2 RESULTS

### 2.1 CAT2 promotes ACX2/3 activity through interaction as well as catalase activity

**FIGURE 1:**
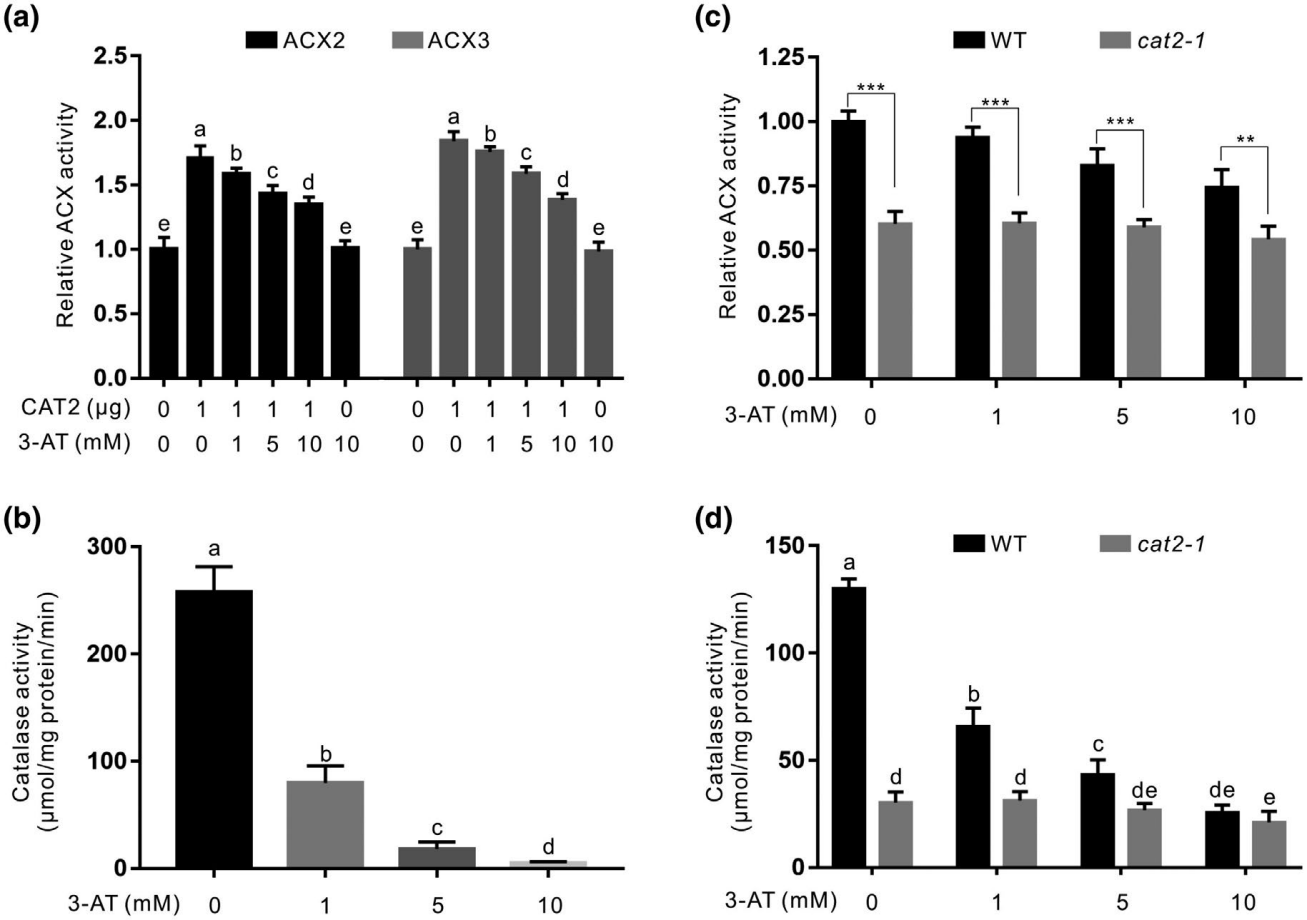
Inhibiting CAT2 activity cannot completely diminish the stimulatory effect of CAT2 on ACX2/3 enzymatic activity. (a) The enzymatic activities of ACX2 and ACX3 were assessed with OPC4‐CoA as the substrate. One microgram of ACX2 or ACX3 protein was added in the reaction buffer containing different concentrations of CAT2 and 3‐AT, and the reaction was incubated at 25 °C for 30 min. As ACX2 and ACX3 convert substrate OPC4‐CoA to product Δ^2^‐OPC4‐CoA, thus the ACX2 andACX3 activities are defined as the ratio of Δ^2^‐OPC4‐CoA to (Δ^2^‐OPC4‐CoA + OPC4‐CoA). The method to detect ACX activity is shown in the Experimental Procedures section. The activity of untreated ACX2 or ACX3 was set to 1. (b) Catalase activity of purified CAT2 protein treated with different concentrations of 3‐AT was assayed. The ACX activity (c) and catalase activity (d) of the untreated and 3‐AT‐treated proteins extracted from the 4‐week‐old wildtype and *cat2‐1* mutant plant were assayed. Data are means (± *SD*) of three biological replicates. Bars with different letters indicate significant differences at *p* < 0.05, revealed using a one‐way analysis of variance with a Tukey's multiple comparison test. Asterisks indicate significant differences using Student's *t* test, *p < 0.05, **p < 0.01, ***p < 0.001

Previously, we reported that CAT2 interacts with and promotes ACX2/3 to enhance JA biosynthesis, thus increasing plant resistance to necrotrophic *B*. *cinerea* B05.10 infection (Yuan et al., 2017), while how CAT2 increases ACX activity is not clear. We previously speculated that CAT2 may promote ACX activity by scavenging H_2_O_2_, one of the products of ACX with fatty acyl‐CoA as the substrate. Indeed, inhibition of CAT2 activity by SA, a catalase inhibitor, significantly represses CAT2‐mediated stimulation of ACX2/3 activity. However, we also found that even high concentrations of SA treatment cannot totally diminish the stimulatory effect of CAT2 on ACX2/3 activity, suggesting a possibility that CAT2 may also promote ACX2/3 activity through its interaction in addition to its H_2_O_2_‐decomposing activity. To investigate this, we expressed and purified ACX2 and ACX3 proteins in *Escherichia coli*, and assayed their ACX activity with OPC4‐CoA in the presence or absence of purified CAT2 protein according to our previously reported method (Yuan et al., 2017). Our results showed that the activity of ACX2 or ACX3 was significantly increased when CAT2 was added (Figure 1a), which is consistent with our previous report (Yuan et al., 2017). In addition, 3‐amino‐1,2,4‐triazole (3‐AT), a widely used strong inhibitor of catalase (Liu et al., 2020b), was used, and we found that 3‐AT dramatically suppressed CAT2 but not ACX2 or ACX3 activity, while 3‐AT markedly repressed CAT2 stimulation of ACX2/3 activity (Figure 1a,b), suggesting that the H_2_O_2_‐decomposing activity of CAT2 functions in promotion of ACX2/3 activity. However, we also found that when CAT2 activity was inhibited to a nearly undetectable level by high concentrations of 3‐AT, ACX2 or ACX3 activity was still significantly higher in the presence of CAT2 than in the absence of CAT2 (Figure 1a,b), suggesting that CAT2 may promote ACX2/3 activity through its interaction as well as catalase activity. To confirm this, total proteins from the wildtype plant and *cat2‐1*, a T‐DNA insertion mutant of *CAT2*, were extracted to examine their ACX activity. Our results showed that 3‐AT inhibited both catalase activity and ACX activity in the wildtype plants in a dosage‐dependent manner, while 10 mM 3‐AT‐treated wildtype plant proteins had similar low catalase activity but still higher ACX activity compared with proteins from the *cat2‐1* mutant (Figure 1c,d), further supporting that CAT2 promotes ACX2/3 activity through its interaction.

**FIGURE 2:**
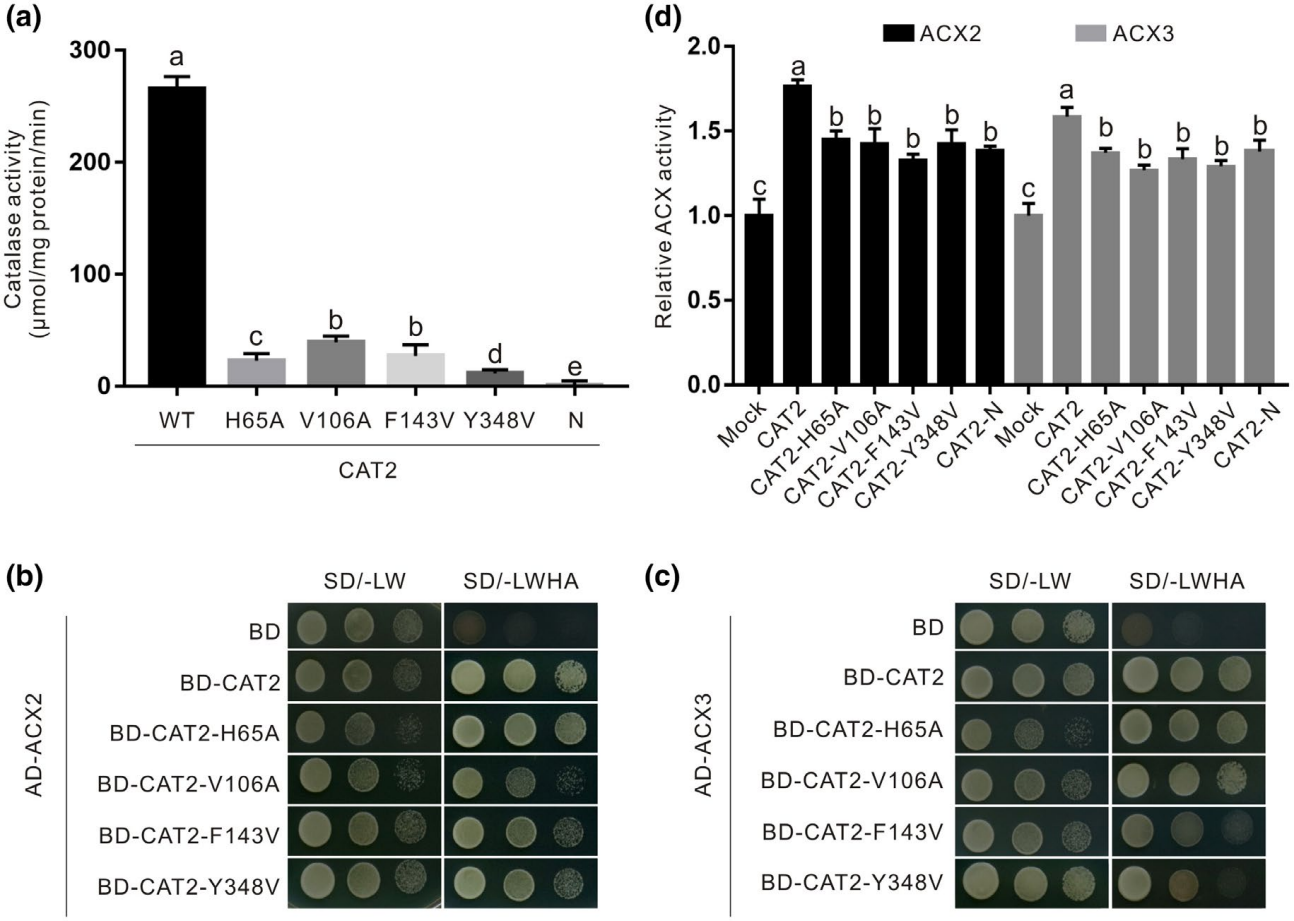
Mutated or truncated N‐terminal CAT2 proteins with low or no catalase activity can interact with and promote ACX2/3 activity. (a) Catalase activity of purified wildtype (WT), mutated, and N‐terminal CAT2 proteins was assayed. The interaction of the wildtype and mutated CAT2 with ACX2 (b) or ACX3 (c) in yeast. (d) The enzymatic activities of ACX2 and ACX3 were assessed with OPC4‐CoA as the substrate in the presence or absence of the WT, mutated, and N‐terminal CAT2 proteins. One microgram of ACX2 or ACX3 protein was added in the reaction buffer with or without 1 µg of CAT2‐H65A, CAT2‐V106A, CAT2‐F143V, CAT2‐Y348V, or CAT2‐N protein, and the reaction was incubated at 25 °C for 30 min, and then OPC4‐CoA and Δ^2^‐OPC4‐CoA in the reaction buffer were measured. Data are means (± *SD*) of three biological replicates. Bars with different letters indicate significant differences at *p* < 0.05, revealed using a one‐way analysis of variance with a Tukey's multiple comparison test

Previous reports documented some key amino acid sites necessary for catalase activity (Fujikawa et al., 2019; Poulos, 2010). If CAT2 really can promote ACX2/3 activity through its interaction and a mutated CAT2 with low or no H_2_O_2_‐decomposing activity can still interact with ACX2 or ACX3, the mutated CAT2 should also stimulate ACX2/3 activity. Therefore, we expressed four mutated CAT2 proteins, CAT2‐H65A, CAT2‐V106A, CAT2‐F143V, and CAT2‐Y348V, according to previous reports (Diaz et al., 2012; Fujikawa et al., 2019; Poulos, 2010). Consistently, the four mutated CAT2 proteins had extremely low catalase activity compared with the wildtype CAT2 (Figure 2a). We performed a yeast two‐hybrid experiment to determine whether these mutations affect CAT2 interaction with ACX2 and ACX3. Our results showed that similar to the wildtype CAT2 protein, CAT2‐H65A, CAT2‐V106A, CAT2‐F143V, and CAT2‐Y348V strongly interacted with ACX2 or ACX3 in yeast (Figure 1b,c). These four mutated CAT2 proteins were used to test their effect on ACX2/3 activity. Interestingly, all four mutated CAT2 proteins promoted the activity of ACX2 or ACX3 (Figure 2d). Together, these results clearly show that CAT2 promotes ACX2/3 activity at least partially through the interaction.

### 2.2 The N‐terminus of CAT2 interacts with and promotes ACX2 and ACX3

**FIGURE 3:**
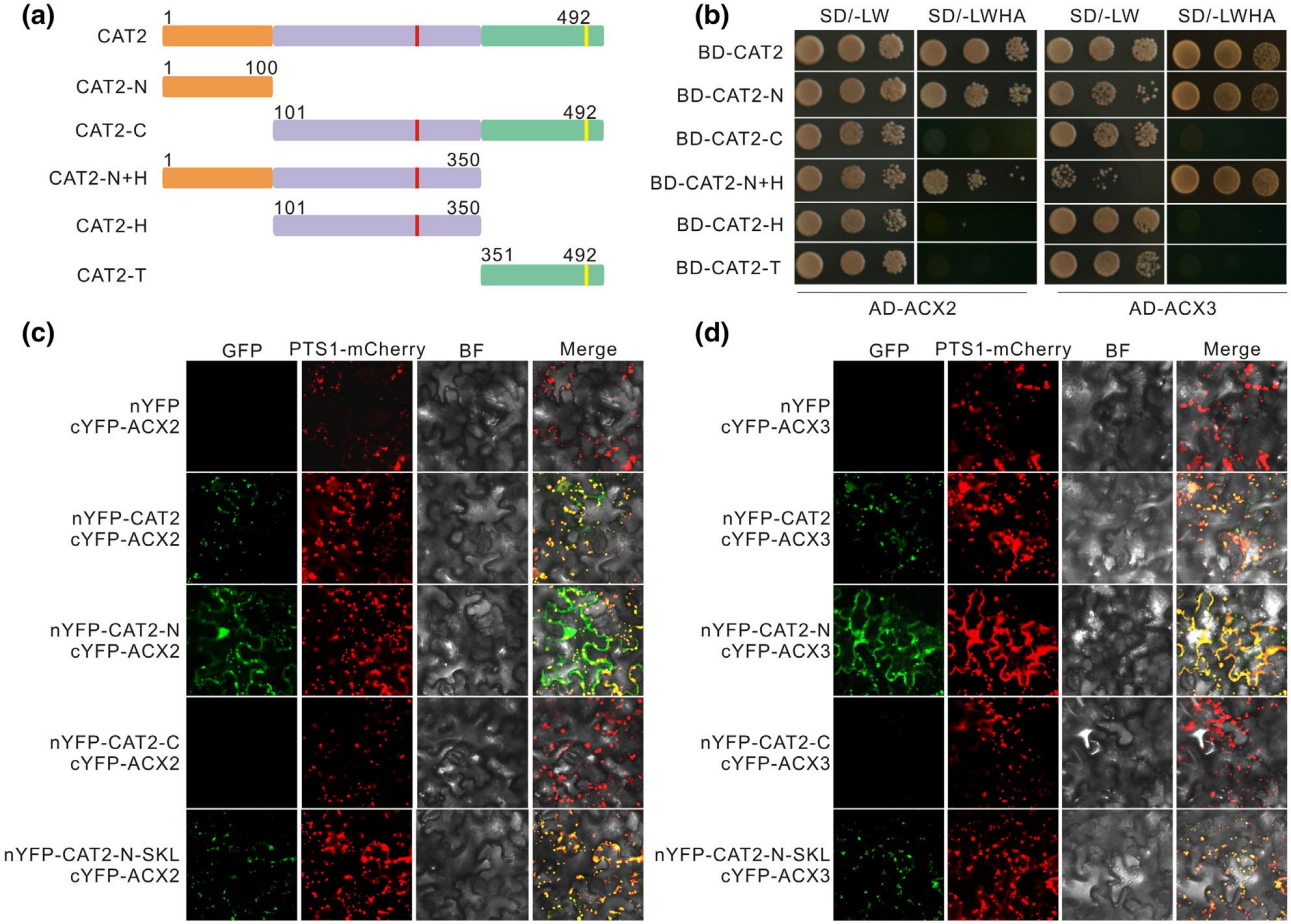
The N‐terminus of CAT2 interacts with ACX2 and ACX3. (a) A schematic representation of the CAT2 protein truncations CAT2‐N, CAT2‐C, CAT2‐H, CAT2‐T, and CAT2‐N+H. The red vertical line in CAT2‐H indicates the predicted heme ligand signature‐containing fragment, and the yellow vertical line in CAT2‐T indicates the peroxisomal targeting signal (PTS) peptide, Q480‐K481‐L482. (b) The interaction of full‐length CAT2 and truncated CAT2 with ACX2 and ACX3 in yeast. Bimolecular fluorescence complementation assay shows the interaction between full‐length CAT2, truncated CAT2, and ACX2 (c) or ACX3 (d) in *Nicotiana benthamiana* leaves. The red fluorescence signal of PTS1‐mCherry was used to indicate the peroxisomes

Protein–protein interaction generally occurs in a small protein interface. To investigate which fragment of CAT2 interacts with and promotes ACX2/3, we analysed *Arabidopsis* CAT2 protein structure according to its homolog protein structure (Poulos, 2010), and divided it into N‐terminus (CAT2‐N) and C‐terminus (CAT2‐C), which was further divided into H‐fragment (heme ligand signature‐containing fragment, CAT2‐H) and T‐fragment (peroxisomal targeting signal peptide‐containing fragment, CAT2‐T) (Figure 3a). The yeast two‐hybrid experiment showed that fragments containing CAT2‐N, including full length CAT2, CAT2‐N, and CAT2‐N+H, could interact with both ACX2 and ACX3, while CAT2‐C, CAT2‐H or CAT2‐T could not (Figure 3b), revealing that the CAT2‐N fragment is required for CAT2–ACX2/3 interaction. Furthermore, we confirmed their interaction by a bimolecular fluorescence complementation (BiFC) experiment in *Nicotiana benthamiana* leaves. Our results showed that a strong reconstituted fluorescence signal was detected in *N*. *benthamiana* leaves coexpressing cYFP‐tagged ACX2 or ACX3 and nYFP‐tagged CAT2‐N but not nYFP‐tagged CAT2‐C (Figure 3c,d), indicating that CAT2‐N interacts with ACX2 and ACX3 in plants. However, we observed that the fluorescence in *N*. *benthamiana* leaves coexpressing cYFP‐tagged ACX2 or ACX3 and nYFP‐tagged CAT2 was mainly in peroxisomes, while the fluorescence was mainly in the cytoplasm for cYFP‐tagged ACX2 or ACX3 and nYFP‐tagged CAT2‐N. This is perhaps due to the lack of a peroxisomal targeting signal (PTS) sequence in CAT2‐N. Thus, we fused a type I PTS sequence, Ser‐Lys‐Leu (SKL) with nYFP‐tagged CAT2‐N, and assayed its interaction with ACX2 and ACX3 by BiFC. Our results showed that coexpressing nYFP‐tagged CAT2‐N‐SKL and cYFP‐tagged ACX2 or ACX3 resulted in significant fluorescence mainly in peroxisomes in *N*. *benthamiana* leaves (Figure 3c,d).

To determine whether the CAT2‐N fragment has H_2_O_2_‐decomposing activity, we expressed and purified CAT2‐N in *E*. *coli* and examined its catalase activity. Our results showed that compared with the full‐length CAT2 protein, CAT2‐N exhibited no catalase activity (Figure 2a). This prompted us to further assay whether CAT2‐N can promote the activity of ACX2 and ACX3 as they have interaction in vitro and in vivo. Therefore, we assessed the activity of ACX2 and ACX3 in the presence or absence of CAT2‐N. Our results showed that the activity of ACX2 and ACX3 was significantly increased as CAT2‐N protein was added (Figure 3b). These data clearly indicate that the N‐terminus of CAT2 interacts with ACX2/3 and promotes their activity.

### 2.3 *CAT2‐N* partially rescues increased susceptibility of *cat2‐1* mutant to *B*. *cinerea* B05.10

**FIGURE 4:**
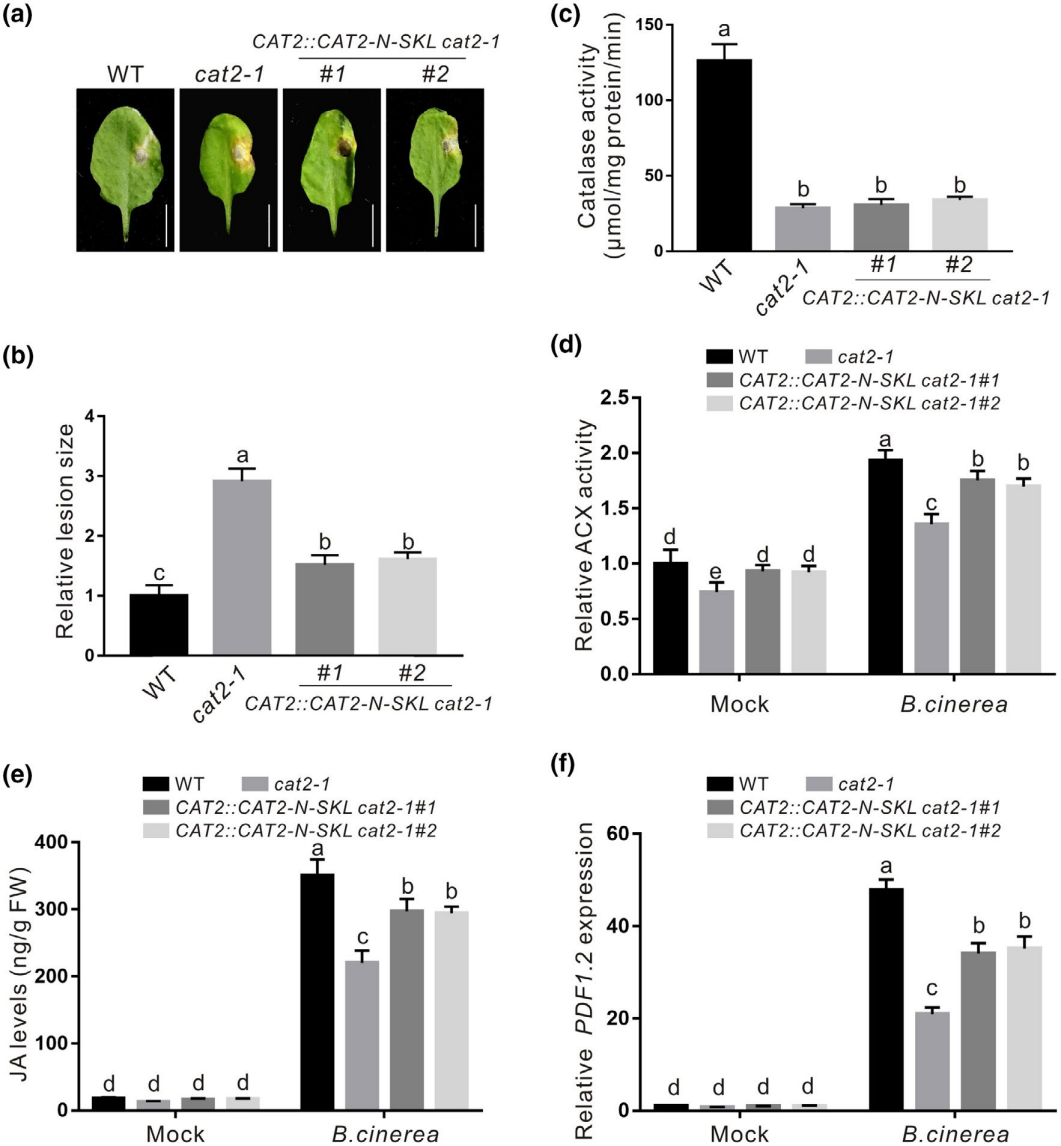
The *CAT2‐N‐SKL* partially rescues decreased resistance of *cat2‐1* mutant to *Botrytis cinerea* B05.10. Photographs of rosette leaves (a) cut from 4‐week‐old wildtype (WT), *cat2‐1*, and *CAT2*::*CAT2‐N‐SKL cat2‐1* transgenic plants at 3 days after infection with *B. cinerea* B05.10 spores. The relative lesion size is shown (b). Data are means (± *SD*) of three biological replicates. Bars with different letters indicate significant differences at *p* < 0.05, revealed using a one‐way analysis of variance (ANOVA) with a Tukey's multiple comparison test. (c) Catalase activity of rosette leaves cut from 4‐week‐old WT, *cat2‐1*, and *CAT2*::*CAT2‐N‐SKL cat2‐1* transgenic plants. Data are means (± *SD*) of three biological replicates. Bars with different letters indicate significant differences at *p* < 0.05, revealed using a one‐way ANOVA with a Tukey's multiple comparison test. The ACX activity (c) and JA levels (d) in the WT, *cat2‐1*, and *CAT2::CAT2‐N‐SKL cat2‐1* transgenic plants infected with or without *B*. *cinerea* B05.10. Data are means (± *SD*) of three biological replicates. Different letters indicate statistically significant differences by two‐way ANOVA with Tukey's post hoc test (*p* < 0.05). (f) The expression of *PDF1.2* was assayed by quantitative reverse transcription PCR in the leaves of the WT, *cat2‐1*, and *CAT2*::*CAT2‐N‐SKL cat2‐1* transgenic plants 3 days after infection with *B*. *cinerea* B05.10. *ACTIN2/8* was used as the reference gene. Data are means (± *SD*) of three biological replicates. Different letters indicate statistically significant differences by two‐way ANOVA with Tukey's post hoc test (*p* < 0.05)

The stimulatory effect of CAT2‐N on ACX2/3 activity in vitro suggests its possible in vivo role in plant resistance to *B. cinerea* B05.10. We therefore generated *CAT2::CAT2‐N‐SKL cat2‐1* transgenic plants in which the expression of *CAT2‐N‐SKL* was driven by the *CAT2* native promoter in the *cat2‐1* mutant, and then tested their resistance to *B. cinerea* B05.10. Our results showed that the *cat2‐1* mutant was susceptible to *B. cinerea* B05.10 compared with the wildtype plant, as we previously reported (Yuan et al., 2017), while the expression of *CAT2‐N‐SKL* driven by the *CAT2* promoter did not alter the catalase activity of the *cat2‐1* mutant but partially rescued the sensitivity of the mutant to infection (Figure 4a,b). Additionally, we examined the ACX activity and JA levels in these transgenic plants, and found that decreased ACX activity and JA level in the *cat2‐1* mutant were also partially increased by the expression of *CAT2‐N‐SKL* (Figure 4c,d). Moreover, we detected the expression of *PDF1.2*, a JA‐responsive marker gene involved in plant response to necrotrophic pathogen infection, in the wildtype, *cat2‐1*, and these transgenic plants. As shown in Figure 4d, lower expression of *PDF1.2* in *B. cinerea‐*infected *cat2‐1* mutant was partly reverted by *CAT2‐N‐SKL*, which is consistent with the effect of *CAT2‐N‐SKL* on ACX activity and JA levels in the mutant. These data clearly indicate that CAT2‐N can promote ACX2/3 activity in vivo to stimulate ACX2/3 activity and JA biosynthesis, thus partially rescuing reduced resistance of the *cat2‐1* mutant to *B. cinerea* B05.10.

### 2.4 *CAT2‐N*‐overexpressing plants have enhanced resistance to *B*. *cinerea* B05.10

**FIGURE 5:**
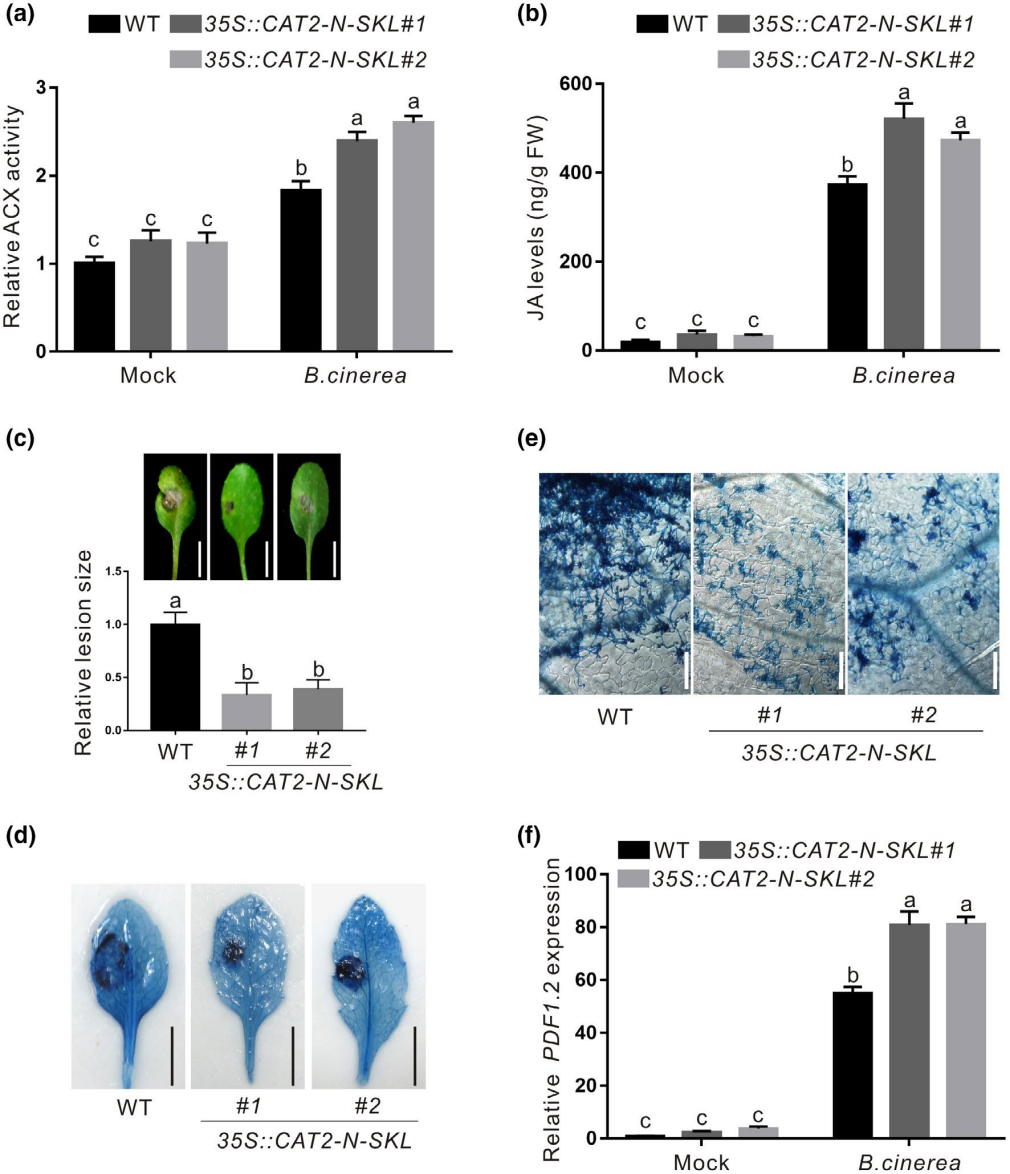
The *CAT2‐N‐SKL*‐overexpressing plants have enhanced resistance to *Botrytis cinerea* B05.10. ACX activity (a) and jasmonic acid (JA) levels (b) in the wildtype (WT) and 35S::*CAT2‐N‐SKL* plants infected with or without *B*. *cinerea* B05.10. The lesion phenotype (c, top), relative lesion size (c, bottom), trypan blue staining images (d), and hyphal growth (e) are shown in the leaves of the WT and 35S::*CAT2‐N‐SKL* plants 3 days after infection with *B*. *cinerea* B05.10. (f) The expression of *PDF1.2* was assayed by quantitative reverse transcription PCR in the leaves of the WT and 35S::*CAT2‐N‐SKL* plants 3 days after infection with *B*. *cinerea* B05.10. *ACTIN2/8* was used as the reference gene. Data are means (± *SD*) of three biological replicates. Different letters indicate statistically significant differences by two‐way analysis of variance with Tukey's post hoc test (*p* < 0.05)

To further confirm the role of CAT2‐N in plant ACX activity, JA biosynthesis, and resistance to *B. cinerea* B05.10, we generated transgenic *Arabidopsis* plants overexpressing *CAT2‐N‐SKL* in the wildtype plant, 35S::*CAT2‐N‐SKL*, and first examined their ACX activity. Our results showed that the 35S::*CAT2‐N‐SKL* plants had significantly higher ACX activity than the wildtype plants when infected with *B. cinerea* B05.10 (Figure 5a). In addition, JA levels were measured in the wildtype and 35S::*CAT2‐N‐SKL* plants infected with or without *B*. *cinerea* B05.10. Our results showed that JA levels in *B*. *cinerea* B05.10‐infected 35S::*CAT2‐N‐SKL* plants were significantly higher than in the infected wildtype plant (Figure 5b). Increased JA levels suggested enhanced resistance to *B. cinerea* B05.10 (Koornneef & Pieterse, 2008; Wasternack & Song, 2017). Thus, we further evaluated the resistance of the wildtype and 35S::*CAT2‐N‐SKL* plants to *B. cinerea* B05.10. We found that the infected 35S::*CAT2‐N‐SKL* plants had smaller lesion size and slower fungal hyphae growth than the wildtype plant (Figure 5c,d,e), indicating that the 35S::*CAT2‐N‐SKL* plants have increased resistance to *B. cinerea* B05.10. Consistently, the expression of *PDF1.2* was extensively higher in *B. cinerea‐*infected 35S::*CAT2‐N‐SKL* plants than the infected wildtype plants (Figure 5f), further supporting that *CAT2‐N*‐overexpressing transgenic plants exhibit enhanced resistance to necrotrophic *B. cinerea* B05.10.

### 2.5 Overexpressing *CAT2‐N* partly releases antagonism of SA on JA

**FIGURE 6:**
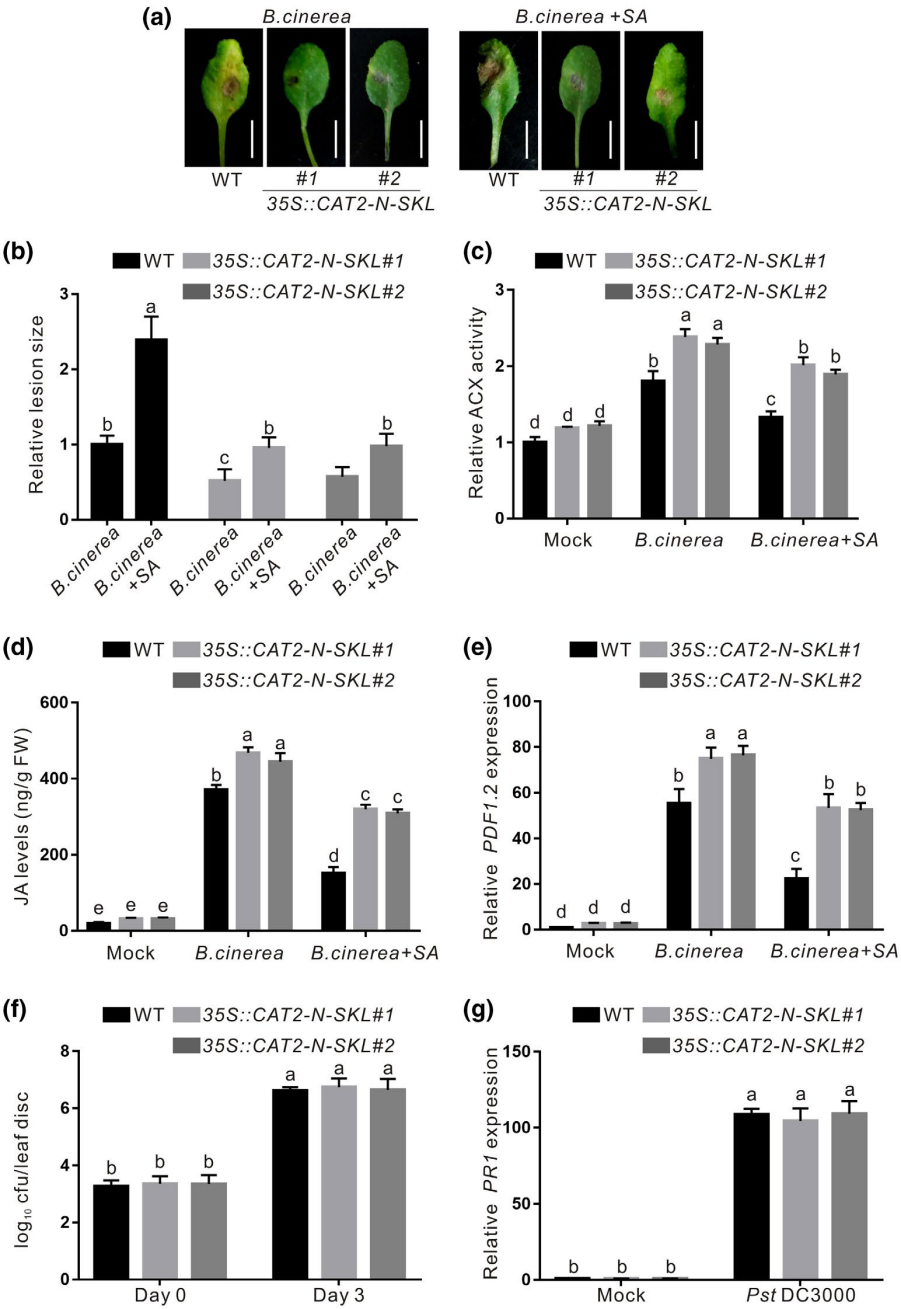
Effect of salicylic acid (SA) on the resistance of the wildtype (WT) and 35S::*CAT2‐N‐SKL* plants to *Botrytis cinerea* B05.10. (a, b) The disease symptoms of the leaves of the WT and 35S::*CAT2‐N‐SKL* plants 3 days after infection with *B*. *cinerea* B05.10 in the presence or absence of SA. Both the WT and 35S::*CAT2‐N‐SKL* plants were pretreated with water or SA for 1 day and then inoculated with *B. cinerea* B05.10. Three days after infection with *B*. *cinerea* B05.10, the photographs of the leaves were taken (a), and the relative lesion size is shown (b). ACX activity (c) and JA levels (d) in leaves of the WT and 35S::*CAT2‐N‐SKL* plants 3 days after infection with *B*. *cinerea* B05.10 in the presence or absence of SA. (e) The expression of *PDF1.2* was assayed by quantitative reverse transcription PCR (RT‐qPCR) in the leaves of the WT and 35S::*CAT2‐N‐SKL* plants 3 days after infection with *B*. *cinerea* B05.10 in the presence or absence of SA. *ACTIN2/8* was used as the reference gene. (f) Bacterial growth was measured at days 0 and 3 after the WT and 35S::*CAT2‐N‐SKL* plants were infected with *Pseudomonas syringae* pv. *tomato* (Pst) DC3000. (g) The expression of *PR1* was assayed by RT‐qPCR in the leaves of the WT and 35S::*CAT2‐N‐SKL* plants 3 days after infection with Pst DC3000. *ACTIN2/8* was used as the reference gene. Data are means (± *SD*) of three biological replicates. Different letters indicate statistically significant differences by two‐way analysis of variance with Tukey's post hoc test (*p* < 0.05)

SA and JA are two crucial phytohormones mediating plant defence responses to biotrophic and necrotrophic pathogens, respectively (Beckers & Spoel, 2006; Koornneef & Pieterse, 2008; Spoel & Dong, 2008; Van der Does et al., 2013). However, SA and JA usually act antagonistically in plant immune responses (Ndamukong et al., 2007; Spoel et al., 2003). Our previous report documented that SA treatment or biotrophic bacterial infection‐induced SA dramatically represses JA accumulation by inhibiting CAT2 activity in plants, resulting in increased susceptibility to necrotrophic *B. cinerea* B05.10 (Yuan et al., 2017). Considering that CAT2‐N has no catalase activity but can still enhance plant resistance to *B. cinerea* B05.10 through promoting ACX2/3 activity and JA biosynthesis, we further dissected the effect of SA on JA biosynthesis and resistance of the wildtype and 35S::*CAT2‐N‐SKL* plants to *B. cinerea*. Consistently, SA‐pretreated wildtype plants are more susceptible to *B. cinerea* B05.10 infection than the unpretreated plants, while the 35S::*CAT2‐N‐SKL* plants were less sensitive to SA when challenged with *B. cinerea* B05.10 in terms of lesion size (Figure 6a). This is because SA markedly repressed *B*. *cinerea* infection‐induced ACX activity and JA accumulation in the wildtype but not in the 35S::*CAT2‐N‐SKL* plants (Figure 6b,c). Also, *B. cinerea* B05.10 infection‐activated defence gene *PDF1.2* expression was significantly suppressed in the wildtype but not in the 35S::*CAT2‐N‐SKL* plants (Figure 6d).

We further assessed whether increased resistance to *B. cinerea* B05.10 of the *CAT2‐N‐*overexpressing plants is sensitive to biotrophic pathogen Pst DC3000 (Figure 6e). We found that the 35S::*CAT2‐N‐SKL* plants exhibited similar resistance to Pst DC3000 with the wildtype plant in terms of bacterial growth and the expression of pathogenesis‐related defence genes *PR1* (Figure 6f), revealing that overexpression of *CAT2‐N* does not affect plant resistance to Pst DC3000.

## 3 DISCUSSION

In this study, we identified CAT2‐N, not CAT2‐C, as an accelerator for JA biosynthesis during plant resistance to *B. cinerea* through interacting with and promoting ACX2/3. In addition, mutation of the crucial active amino acids of CAT2, including His65, Val106, Phe143, and Tyr348, resulted in extremely low catalase activity for H_2_O_2_ degradation, while these mutated CAT2 proteins still interacted with ACX2 and ACX3, and stimulated their ACX activity (Figure 2), further supporting that CAT2 promotes ACX activity at least partially dependent on protein–protein interaction. We speculate that CAT2 or CAT2‐N may stimulate ACX activity by changing the conformation of ACX2 and ACX3 or serving as their molecular chaperone. Thus, resolving the co‐crystallization structure of the CAT2–ACX2/3 protein complex or examining the effect of CAT2 on ACX2/3 protein stability in plants may shed some light on the underlying mechanism.

A recent report showed that a loss‐of‐function mutant of *AtIQM1* (*IQ‐Motif Containing Protein1*), *iqm1‐1*, exhibited higher resistance to biotrophic Pst DC3000 but susceptibility to necrotrophic *B*. *cinerea* B05.10 than the wildtype plant (Lv et al., 2019). AtIQM1 interacts with and promotes CAT2 activity, especially in the presence of calmodulin5 (CaM5) in *Arabidopsis*, increasing the activity of the JA biosynthetic enzymes ACX2 and ACX3 (Lv et al., 2019). In this study, we found that overexpressing *CAT2‐N* in the wildtype plants did not significantly alter plant resistance to Pst DC3000 but enhanced plant resistance to *B*. *cinerea* B05.10. It might be worth generating transgenic crops with increased *CAT2‐N* expression but decreased *IQM1* expression, as they may have higher resistance to both biotrophic Pst DC3000 and necrotrophic *B*. *cinerea* B05.10.

CAT2 is synthesized in the cytoplasm and imported into peroxisomes based on its C‐terminal atypical PTS1, Q480‐K481‐L482 (Fujikawa et al., 2019). The truncated N‐terminus of CAT2 cannot be properly localized in peroxisomes due to a lack of PTS, thus the interaction between CAT2 and ACX2/3 was in the cytoplasm, as shown in our results (Figure 3c,d). Even though CAT2‐N can promote ACX2/3 activity in vitro, we speculated that CAT2‐N without a PTS sequence fails to stimulate ACX activity in planta because ACX2 and ACX3 are peroxisome‐localized proteins and their substrates, fatty acyl‐CoA, are utilized in peroxisomes. In our study, a typical PTS1, SKL, was fused with the C‐terminal of CAT2‐N fragment, guaranteeing its peroxisomal localization and possible function. As expected, the interaction between CAT2‐N and ACX2/3 was in peroxisomes (Figure 3c,d), and transgenic plants with overexpression of *CAT2‐N‐SKL* had increased ACX activity and JA accumulation in response to *B*. *cinerea* B05.10 infection (Figure 5a,b). It is worth exploring the role of *Arabidopsis* CAT2‐N in other plants, especially the related species such as *Brassica rapa* and *Raphanus sativus*, and potentially improve their resistance to necrotrophic pathogens by genetically manipulating *CAT2‐N*.

Early reports showed that SA can bind to catalases in several plant species, including *Arabidopsis* and tobacco, and inhibit their H_2_O_2_‐decomposing activity (Chen et al., 1993a, 1993b; Pokotylo et al., 2019; Sanchez‐Casas & Klessig, 1994). We also reported that SA inhibits CAT2 to repress *B. cinerea* B05.10‐induced ACX activity for JA synthesis (Yuan et al., 2017). In this study, we found that CAT2‐N with no catalase activity interacted with and promoted ACX2/3, raising the possibility that antagonism of SA on JA biosynthesis would be diminished in plants with overexpression of *CAT2‐N*. Indeed, the 35S::*CAT2‐N‐SKL* plants showed less sensitivity to SA than the wildtype plant, evidenced by higher ACX activity, JA levels, and resistance to *B. cinerea* B05.10 (Figure 6). These results also suggest that SA does not affect the interaction between CAT2‐N and ACX2/3. However, which fragment of CAT2 has the SA‐binding sites remains unknown and is worthy of further exploration.

In summary, our study reveals that CAT2‐N interacts with and promotes ACX2/3 implicated in JA biosynthesis, and thus can be utilized to enhance plant resistance to *B*. *cinerea*. This truncated protein also partly relieves SA repression of JA biosynthesis during plant defence response to *B. cinerea* B05.10.

## 4 EXPERIMENTAL PROCEDURES

### 4.1 Plant material and growth conditions

*Arabidopsis thaliana* ecotype Columbia was used in this study. The wildtype, *cat2‐1* (SALK_076998), *CAT2*::*CAT2‐N‐SKL cat2‐1*, and 35S::*CAT2‐N‐SKL* plants were grown in soil at 22 °C under 16/8 hr day/night cycles, and the intensity of the light was set at 150 µmol⋅m^−2^⋅s^−1^.

### 4.2 Yeast two‐hybrid assay

The plasmids pGBKT7‐CAT2, pGADT7‐ACX2, and pGADT7‐ACX3 were as previously reported (Yuan et al., 2017). The mutated *CAT2*, *CAT2‐H65A*, *CAT2‐V106A*, *CAT2‐F143V*, and *CAT2‐Y348V* were amplified by PCR‐based side‐directed mutagenesis and cloned into pGBKT7 vector at the *Bam*HI site. The truncated *CAT2*, *CAT2‐N*, *CAT2‐H*, *CAT2‐T*, and *CAT2N+H* were amplified by PCR from the full‐length coding sequence of *CAT2*. The yeast transformation and growth were carried out according to our previous report (Yuan et al., 2017). Primer sequences are listed in Table S1.

### 4.3 Protein expression and purification

The prokaryotic expression plasmids pET28a‐CAT2, pET28a‐ACX2, and pET28a‐ACX3 were as previously reported (Yuan et al., 2017). To prepare the proteins of mutated CAT2 and truncated CAT2, the coding sequences of mutated *CAT2* and truncated *CAT2* were cloned into the prokaryotic expression vector pET28a at the *Bam*HI site, and then transformed into *E. coli* BL21 (DE3). The expression and purification of the proteins were performed according to our previous reports (Yuan et al., 2017; Zhang et al., 2020d).

### 4.4 Detection of catalase (CAT) activity

The treated or untreated plants were ground to fine powder under liquid nitrogen, and suspended in freshly prepared, cold, protein extraction buffer (50 mM potassium phosphate buffer, pH 7.8, 0.2 mM EDTA‐Na_2_, 0.1 mM ascorbic acid, 1% polyvinylpolypyrrolidone [PVPP]). After centrifugation at 12,000 × g for 10 min at 4 °C, the supernatant was transferred to a new tube for further use. The protein concentration was assayed by the Bradford method, and CAT activity was determined according to the published methods (Aebi, 1984; Li et al., 2020a). CAT activity was assayed by monitoring the consumption of H_2_O_2_ at 240 nm. To determine the effect of 3‐AT on catalase activity, different concentrations of 3‐AT were treated with the purified CAT2 proteins, and then the catalase activity was assayed according to the above method.

### 4.5 Bimolecular fluorescence complementation

For the bimolecular fluorescence complementation (BiFC) assays, the coding sequence of *Arabidopsis* CAT2 and truncated CAT2 were cloned into pSP‐YNE, and the coding sequences of ACX2 and ACX3 were cloned into pSP‐YCE. To label the peroxisomes in *N. benthamiana* leaves, mCherry fused with the SKL sequence were amplified from the PTS‐RFP plasmid as we previously reported (Yuan et al., 2017), and cloned into pEGAD vector at the *Age*I and *Eco*RI sites, resulting in 35S::mCherry‐SKL.

The resultant plasmids were introduced into *Agrobacterium tumefaciens* GV3101 and coinfiltrated the *N. benthamiana* leaves. Three days after infiltration, the leaves were used to observe the YFP and mCherry fluorescent signals under a confocal laser scanning microscope (Carl Zeiss LSM710 META laser scanning microscope).

### 4.6 Quantitative PCR

Treated or untreated plant leaves were collected for total RNA isolation, first‐strand cDNA synthesis, and quantitative reverse transcription PCR (RT‐qPCR) as described previously (Liu et al., 2018). The constitutively expressed *ACTIN2/8* gene was used as an internal control. All experiments were repeated at least three times. The primer sequences are listed in Table S1.

### 4.7 Construction of *CAT2*::*CAT2‐N‐SKL cat2‐1* and 35S::*CAT2‐N‐SKL* transgenic plants

The genomic sequence of 1,997 bp upstream of the *CAT2* translation start codon (ATG) and the coding sequence of *CAT2‐N* fused with the sequence of SKL (TCGAAGCTG) were amplified and cloned into pCAMBIA1300 between the *Eco*RI/*Sac*I and *Kpn*I/*Pst*I restriction sites, respectively. The resulting plasmid pCAMBIA1300‐CAT2::CAT2‐N‐SKL was introduced into the *cat2‐1* mutant via *A*. *tumefaciens*‐mediated floral transformation.

The coding sequence of *CAT2‐N* fused with the sequence of SKL (TCGAAGCTG) was amplified by PCR and inserted into pEGAD vector at the *Age*I and *Eco*RI sites, resulting in 35S::CAT2‐N‐SKL. The resultant plasmid was introduced into wildtype *Arabidopsis* by *A*. *tumefaciens* GV3101‐mediated transformation using the floral dip method. All the experiments were conducted with homozygous T_4_ plants.

### 4.8 Plant inoculation

Plant inoculation with *B. cinerea* and Pst DC3000 was carried out according to our previous reported method (Yuan et al., 2017). For *B. cinerea* infection, *B. cinerea* strain B05.10 was cultured and inoculum was prepared as previously described (Song et al., 2014). The spore suspension at a density of 10^6^/ml was prepared, and 10 μl of the suspended spores was used to inoculate the 4‐week‐old plant leaves. Images of the infected plant leaves were captured 3 days after inoculation, and the relative lesion size was analysed according to our previously reported method (Yuan et al., 2017). Infected and uninfected leaves were further used for ACX activity, JA levels, and RT‐qPCR. For Pst DC3000 infection, the virulent strain Pst DC3000 was cultured in King's B medium containing 50 μg/ml rifampicin at 28 °C. Four‐week‐old plant leaves were sprayed with Pst DC3000 (OD_600_ = 0.01) suspended in 10 mM MgCl_2_ and 0.02% Silwet. The bacterial growth was determined at 3 days after inoculation.

### 4.9 Detection of ACX activity

Detection of ACX activity of the plants or purified proteins was performed according to our previously reported method (Liu et al., 2017; Yuan et al., 2017). Briefly, OPC4‐CoA was synthetized using OPC4 purchased from OlChemIm, and CoA and yeast acyl‐CoA synthetase from a test kit (Roche) as previously reported (Li et al., 2005; Yuan et al., 2017). Purified or extracted plant protein was added to the reaction buffer (OPC4‐CoA, 50 mM KPO_4_, pH 7.6, 50 µM flavin adenine dinucleotide [FAD], 100 µg/ml bovine serum albumen [BSA]). After incubation at 25 °C for 30 min, the reaction mixtures were diluted with 100 volumes of 0.1% trifluoroacetic acid [TFA] in acetonitrile, and 1 µl of samples was applied to the MALDI‐TOF/TOF mass spectrometer (AB SCIEX 4800 MALDI‐TOF/TOF) in positive ion mode. Full‐scan mass spectra were obtained by scanning from *m*/*z* 800 to 1,200. Data analysis was carried out using Data Explorer v. 4.3 processing software. The substrate of ACX2/3, OPC4‐CoA, was represented in the spectrum by the molecular ions [OPC4‐CoA+H] ^+^ (*m*/*z* 988.17), [OPC4‐CoA+Na] ^+^ (*m*/*z* 1,010.14) and [OPC4‐CoA+K] ^+^ (*m*/*z* 1,026.23), and the product of ACX2/3 with OPC4‐CoA as the substrate, Δ^2^‐OPC4‐CoA, by the molecular ions [Δ^2^‐OPC4‐CoA+H] ^+^ (*m*/*z* 986.17), [Δ^2^‐OPC4‐CoA+Na] ^+^ (*m*/*z* 1,008.14), and [Δ^2^‐OPC4‐CoA+K]^+^ (*m*/*z* 1,024.13). The ratio of Δ^2^‐OPC4‐CoA to (Δ^2^‐OPC4‐CoA +OPC4‐CoA) is defined as the relative ACX activity.

To examine the ACX activity in plants, infected or uninfected plant leaves were ground to fine powder under liquid nitrogen and then resuspended in cold extraction buffer (50 mM KPO_4_, pH 7.6, 50 mM FAD, 100 µg/ml BSA). After centrifugation at 12,000 × g at 4 °C for 15 min, the supernatant was used to assay the ACX activity.

### 4.10 Trypan blue staining

Trypan blue staining experiments were performed as described previously (Song et al., 2014; Yuan et al., 2017). Briefly, infected plant leaves were soaked in lactophenol‐trypan blue staining solution (0.05% trypan blue, 25% lactic acid, 25% water‐saturated phenol, 50% ethanol) at 37 °C for 1 hr. Chloral hydrate solution (2.5 g/ml) was used to wash the leaves to reduce the background. Images of trypan blue‐stained leaves were captured by a camera, and the leaves were also observed under a microscope.

### 4.11 Measurement of JA levels

JA levels were detected according to our previous reported methods (Yuan et al., 2017; Zhang et al., 2020d). Briefly, the infected and uninfected plant leaves were collected and ground into fine powder under liquid nitrogen. Dihydroxyjasmonic acid (DHJA, 20 ng) was added as an internal standard for JA. The purified samples were then methylated by diazomethane, resuspended in 100 µl of ethyl acetate, and analysed by gas chromatography‐mass spectrometry‐selected ion monitoring (GC/MS‐SIM) (Trace GC Ultra/ISQ; Thermo Fisher Scientific). The compounds were separated on an Rtx‐5MS (30 m × 0.25 mm × 0.25 mm) column. The chromatographic parameters were as follows: 40 °C for 1 min after injection, followed by sequential temperature ramps of 25 °C/min to 150 °C, 5 °C/min to 200 °C, 10 °C/min ramp to 240 °C, and 240 °C for 10 min. The monitored ions were *m*/*z* 151 and 153 for JA and DHJA, respectively.

## CONFLICTS OF INTEREST

All the authors declare that they have no competing interests.

## Supporting information

**TABLE S1** List of the primers used in this study

**TABLE S2** Tukey’s multiple comparisons test results in this study

**TABLE S3** Two‐way analysis of variance in this study

## Data Availability

The data that support the findings of this study are available from the corresponding author upon reasonable request.
