# Supplementary material for: Overexpressing the N‐terminus of CATALASE2 enhances plant jasmonic acid biosynthesis and resistance to necrotrophic pathogen Botrytis cinerea B05.10
Source: Mol Plant Pathol. 2021 Jul 10;22(10):1226–38. doi: 10.1111/mpp.13106 (PMC8435237; doi:10.1111/mpp.13106)
Supplement: Supplementary file 1 — TABLE S1 List of the primers used in this study [file MPP-22-1226-s002.docx]

**Supplemental Table S1**

List of the primers used in this study.

**Primer name Sequence (5’ to 3’)**

**Primers used for molecular cloning**

CAT2-H65A-F  CCAGAGCGTGTGGTTGCTGCCAGAGGAGCCAG

CAT2-H65A-R CTGGCTCCTCTGGCAGCAACCACACGCTCTGG

CAT2-V106A-F CCGGTTCTCCACCGCTATCCATGAGCGTGGAAG

CAT2-V106A-R CTTCCACGCTCATGGATAGCGGTGGAGAACCGG

CAT2-F143V-F CAACTTTCCTGTTGTCTTCATCCGCGATGGG

CAT2-F143V-R CCCATCGCGGATGAAGACAACAGGAAAGTTG

CAT2-Y348V-F  CCCGTGTCTTCTCCGTTGCCGATACTCAGAGAC

CAT2-Y348V-R GTCTCTGAGTATCGGCAACGGAGAAGACACGGG

pET28-CAT2-N-F AGCAAATGGGTCGCGGAATGGATCCTTACAAGTATCGTCC

pET28-CAT2-N-R CGGAGCTCGAATTCGGAAATGACAGGAGTCTGAACACCGG

pgbk-CAT2-N-F GGCCGAATTCCCGGGGATGGATCCTTACAAGTATCGTCC

pgbk-CAT2-N-R CGCTGCAGGTCGACGAATGACAGGAGTCTGAACACCGG

pgbk-CAT2-C-F GGCCGAATTCCCGGGGACTCAGAGACACCGTCTTGGAC

pgbk-CAT2-C-R CGCTGCAGGTCGACGGATGCTTGGTCTCACGTTCAGA

pgbk-CAT2-H-F GGCCGAATTCCCGGGGGTCCGGTTCTCCACCGTTATCC

pgbk-CAT2-H-R CGCTGCAGGTCGACGATCGGCATAGGAGAAGACACG

Pegad-CAT2-N-SKL-f GACTCTAGCGCTACCGGTATGGATCCTTACAAGTATCGTCC

Pegad-CAT2-N-SKL-R CTCGAGCCCGGGGAATTCcagcttcgaAATGACAGGAGTCTGAA

CACC

CAT2pro-F TGACATGATTACGAATTCagtaatcgatcatccttaagtttg

cat2pro-R GAGCTCGTCCCCCGTGTTggtttgatgagaagagagcttg

cat2-F GGTACCCGGGGATCCTCTATGGATCCTTACAAGTATCGTCC

cat2-N-SKL-R CTGCAGTTACTTGTACAGcagcttcgaAATGACAGGAGTCT

GAACACC

YNE-CAT2-N-f GGCGCGCCACTAGTGGATCCATGGATCCTTACAAGTATCGTCC

YNE-CAT2-N-R ACAGTACTATCGATGGATCCAATGACAGGAGTCTGAACACCGG

YNE-CAT2-N-SKL-R ACAGTACTATCGATGGATCCcagcttcgaAATGACAGGAGTCT

GAACACC

YNE-CAT2-C-F GGCGCGCCACTAGTGGATCCACTCAGAGACACCGTCTTGGAC

YNE-CAT2-C-R ACAGTACTATCGATGGATCCGATGCTTGGTCTCACGTTCAGA

Pegad-mCherry-SKL-F GACTCTAGCGCTACCGGTatggtgagcaagggcgaggag

Pegad-mCherry-SKL-R CTCGAGCCCGGGGAATTCcagcttcgatctcttgtacagct

**Primers used for qRT-PCR**

PDF1.2-QF TGCAAGAATCAGTGCATTAACC

PDF1.2-QF ACATGGGACGTAACAGATACAC

PR1-QF ATGAATTTTACTGGCTATTC

PR1-QR AACCCACATGTTCACGGCGGA

ACTIN2/8-F GGTAACATTGTGCTCAGTGGTGG

ACTIN2/8-R AACGACCTTAATCTTCATGCTGC
