# Supplementary material for: Overexpressing the N‐terminus of CATALASE2 enhances plant jasmonic acid biosynthesis and resistance to necrotrophic pathogen Botrytis cinerea B05.10
Source: Mol Plant Pathol. 2021 Jul 10;22(10):1226–38. doi: 10.1111/mpp.13106 (PMC8435237; doi:10.1111/mpp.13106)
Supplement: Supplementary file 2 — TABLE S2 Tukey’s multiple comparisons test results in this study [file MPP-22-1226-s003.docx]

**Table S2.** Tukey's multiple comparisons test results in this study.

**Figure 4d**

| **Tukey's multiple comparisons test** | **Mean Diff.** | **95.00% CI of diff.** | **Significant?** | ***p* Value** |
| --- | --- | --- | --- | --- |
| Mock:WT **vs.** Mock:cat2-1 | 0.2578 | 0.01156 to 0.504 | Yes | 0.0368 |
| Mock:WT **vs.** Mock:CAT2::CAT2N cat2-1#1 | 0.06797 | -0.1783 to 0.3142 | No | 0.9747 |
| Mock:WT **vs.** Mock:CAT2::CAT2N cat2-1#2 | 0.0782 | -0.168 to 0.3244 | No | 0.9478 |
| Mock:WT **vs.** B.c:WT | -0.9319 | -1.178 to -0.6857 | Yes | <0.0001 |
| Mock:WT **vs.** B.c:cat2-1 | -0.3542 | -0.6004 to -0.108 | Yes | 0.0027 |
| Mock:WT **vs.** B.c:CAT2::CAT2N cat2-1#1 | -0.7542 | -1 to -0.508 | Yes | <0.0001 |
| Mock:WT **vs.** B.c:CAT2::CAT2N cat2-1#2 | -0.698 | -0.9443 to -0.4518 | Yes | <0.0001 |
| Mock:cat2-1 **vs.** Mock:CAT2::CAT2N cat2-1#1 | -0.1898 | -0.4361 to 0.05641 | No | 0.2017 |
| Mock:cat2-1 **vs.** Mock:CAT2::CAT2N cat2-1#2 | -0.1796 | -0.4258 to 0.06664 | No | 0.2525 |
| Mock:cat2-1 **vs.** B.c:WT | -1.19 | -1.436 to -0.9435 | Yes | <0.0001 |
| Mock:cat2-1 **vs.** B.c:cat2-1 | -0.612 | -0.8582 to -0.3658 | Yes | <0.0001 |
| Mock:cat2-1 **vs.** B.c:CAT2::CAT2N cat2-1#1 | -1.012 | -1.258 to -0.7658 | Yes | <0.0001 |
| Mock:cat2-1 **vs.** B.c:CAT2::CAT2N cat2-1#2 | -0.9558 | -1.202 to -0.7096 | Yes | <0.0001 |
| Mock:CAT2::CAT2N cat2-1#1 **vs.** Mock:CAT2::CAT2N cat2-1#2 | 0.01023 | -0.236 to 0.2565 | No | >0.9999 |
| Mock:CAT2::CAT2N cat2-1#1 **vs.** B.c:WT | -0.9999 | -1.246 to -0.7537 | Yes | <0.0001 |
| Mock:CAT2::CAT2N cat2-1#1 **vs.** B.c:cat2-1 | -0.4222 | -0.6684 to -0.1759 | Yes | 0.0004 |
| Mock:CAT2::CAT2N cat2-1#1 **vs.** B.c:CAT2::CAT2N cat2-1#1 | -0.8222 | -1.068 to -0.5759 | Yes | <0.0001 |
| Mock:CAT2::CAT2N cat2-1#1 **vs.** B.c:CAT2::CAT2N cat2-1#2 | -0.766 | -1.012 to -0.5198 | Yes | <0.0001 |
| Mock:CAT2::CAT2N cat2-1#2 **vs.** B.c:WT | -1.01 | -1.256 to -0.7639 | Yes | <0.0001 |
| Mock:CAT2::CAT2N cat2-1#2 **vs.** B.c:cat2-1 | -0.4324 | -0.6786 to -0.1862 | Yes | 0.0003 |
| Mock:CAT2::CAT2N cat2-1#2 **vs.** B.c:CAT2::CAT2N cat2-1#1 | -0.8324 | -1.079 to -0.5862 | Yes | <0.0001 |
| Mock:CAT2::CAT2N cat2-1#2 **vs.** B.c:CAT2::CAT2N cat2-1#2 | -0.7762 | -1.022 to -0.53 | Yes | <0.0001 |
| B.c:WT **vs.** B.c:cat2-1 | 0.5777 | 0.3315 to 0.824 | Yes | <0.0001 |
| B.c:WT **vs.** B.c:CAT2::CAT2N cat2-1#1 | 0.1777 | -0.06851 to 0.424 | No | 0.2628 |
| B.c:WT **vs.** B.c:CAT2::CAT2N cat2-1#2 | 0.2339 | -0.01234 to 0.4801 | No | 0.069 |
| B.c:cat2-1 **vs.** B.c:CAT2::CAT2N cat2-1#1 | -0.4 | -0.6462 to -0.1538 | Yes | 0.0008 |
| B.c:cat2-1 **vs.** B.c:CAT2::CAT2N cat2-1#2 | -0.3438 | -0.5901 to -0.09759 | Yes | 0.0035 |
| B.c:CAT2::CAT2N cat2-1#1 **vs.** B.c:CAT2::CAT2N cat2-1#2 | 0.05617 | -0.1901 to 0.3024 | No | 0.9913 |

**Figure 4e**

| **Tukey's multiple comparisons test** | **Mean Diff.** | **95.00% CI of diff.** | **Significant?** | ***p* Value** |
| --- | --- | --- | --- | --- |
| Mock:WT **vs.** Mock:cat2-1 | 4.832 | -31.61 to 41.27 | No | 0.9997 |
| Mock:WT **vs.** Mock:CAT2::CAT2N cat2-1#1 | 1.281 | -35.16 to 37.72 | No | >0.9999 |
| Mock:WT **vs.** Mock:CAT2::CAT2N cat2-1#2 | 0.6644 | -35.78 to 37.11 | No | >0.9999 |
| Mock:WT **vs.** B.c:WT | -332.5 | -369 to -296.1 | Yes | <0.0001 |
| Mock:WT **vs.** B.c:cat2-1 | -202.6 | -239 to -166.1 | Yes | <0.0001 |
| Mock:WT **vs.** B.c:CAT2::CAT2N cat2-1#1 | -279.8 | -316.3 to -243.4 | Yes | <0.0001 |
| Mock:WT **vs.** B.c:CAT2::CAT2N cat2-1#2 | -276.6 | -313.1 to -240.2 | Yes | <0.0001 |
| Mock:cat2-1 **vs.** Mock:CAT2::CAT2N cat2-1#1 | -3.551 | -39.99 to 32.89 | No | >0.9999 |
| Mock:cat2-1 **vs.** Mock:CAT2::CAT2N cat2-1#2 | -4.168 | -40.61 to 32.27 | No | 0.9999 |
| Mock:cat2-1 **vs.** B.c:WT | -337.4 | -373.8 to -300.9 | Yes | <0.0001 |
| Mock:cat2-1 **vs.** B.c:cat2-1 | -207.4 | -243.8 to -171 | Yes | <0.0001 |
| Mock:cat2-1 **vs.** B.c:CAT2::CAT2N cat2-1#1 | -284.6 | -321.1 to -248.2 | Yes | <0.0001 |
| Mock:cat2-1 **vs.** B.c:CAT2::CAT2N cat2-1#2 | -281.4 | -317.9 to -245 | Yes | <0.0001 |
| Mock:CAT2::CAT2N cat2-1#1 **vs.** Mock:CAT2::CAT2N cat2-1#2 | -0.6166 | -37.06 to 35.82 | No | >0.9999 |
| Mock:CAT2::CAT2N cat2-1#1 **vs.** B.c:WT | -333.8 | -370.3 to -297.4 | Yes | <0.0001 |
| Mock:CAT2::CAT2N cat2-1#1 **vs.** B.c:cat2-1 | -203.8 | -240.3 to -167.4 | Yes | <0.0001 |
| Mock:CAT2::CAT2N cat2-1#1 **vs.** B.c:CAT2::CAT2N cat2-1#1 | -281.1 | -317.5 to -244.7 | Yes | <0.0001 |
| Mock:CAT2::CAT2N cat2-1#1 **vs.** B.c:CAT2::CAT2N cat2-1#2 | -277.9 | -314.3 to -241.5 | Yes | <0.0001 |
| Mock:CAT2::CAT2N cat2-1#2 **vs.** B.c:WT | -333.2 | -369.7 to -296.8 | Yes | <0.0001 |
| Mock:CAT2::CAT2N cat2-1#2 **vs.** B.c:cat2-1 | -203.2 | -239.7 to -166.8 | Yes | <0.0001 |
| Mock:CAT2::CAT2N cat2-1#2 **vs.** B.c:CAT2::CAT2N cat2-1#1 | -280.5 | -316.9 to -244 | Yes | <0.0001 |
| Mock:CAT2::CAT2N cat2-1#2 **vs.** B.c:CAT2::CAT2N cat2-1#2 | -277.3 | -313.7 to -240.8 | Yes | <0.0001 |
| B.c:WT **vs.** B.c:cat2-1 | 130 | 93.55 to 166.4 | Yes | <0.0001 |
| B.c:WT **vs.** B.c:CAT2::CAT2N cat2-1#1 | 52.73 | 16.29 to 89.17 | Yes | 0.0025 |
| B.c:WT **vs.** B.c:CAT2::CAT2N cat2-1#2 | 55.94 | 19.5 to 92.38 | Yes | 0.0014 |
| B.c:cat2-1 **vs.** B.c:CAT2::CAT2N cat2-1#1 | -77.26 | -113.7 to -40.81 | Yes | <0.0001 |
| B.c:cat2-1 **vs.** B.c:CAT2::CAT2N cat2-1#2 | -74.05 | -110.5 to -37.61 | Yes | <0.0001 |
| B.c:CAT2::CAT2N cat2-1#1 **vs.** B.c:CAT2::CAT2N cat2-1#2 | 3.205 | -33.24 to 39.65 | No | >0.9999 |

**Figure 4f**

| **Tukey's multiple comparisons test** | **Mean Diff.** | **95.00% CI of diff.** | **Significant?** | ***p* Value** |
| --- | --- | --- | --- | --- |
| Mock:WT **vs.** Mock:cat2-1 | 0.2289 | -4.143 to 4.601 | No | >0.9999 |
| Mock:WT **vs.** Mock:CAT2::CAT2N cat2-1#1 | 0.01443 | -4.358 to 4.387 | No | >0.9999 |
| Mock:WT **vs.** Mock:CAT2::CAT2N cat2-1#2 | 0.01957 | -4.353 to 4.392 | No | >0.9999 |
| Mock:WT **vs.** B.c:WT | -46.83 | -51.2 to -42.46 | Yes | <0.0001 |
| Mock:WT **vs.** B.c:cat2-1 | -19.98 | -24.36 to -15.61 | Yes | <0.0001 |
| Mock:WT **vs.** B.c:CAT2::CAT2N cat2-1#1 | -33.09 | -37.46 to -28.72 | Yes | <0.0001 |
| Mock:WT **vs.** B.c:CAT2::CAT2N cat2-1#2 | -34.18 | -38.55 to -29.81 | Yes | <0.0001 |
| Mock:cat2-1 **vs.** Mock:CAT2::CAT2N cat2-1#1 | -0.2145 | -4.587 to 4.158 | No | >0.9999 |
| Mock:cat2-1 **vs.** Mock:CAT2::CAT2N cat2-1#2 | -0.2094 | -4.582 to 4.163 | No | >0.9999 |
| Mock:cat2-1 **vs.** B.c:WT | -47.06 | -51.43 to -42.69 | Yes | <0.0001 |
| Mock:cat2-1 **vs.** B.c:cat2-1 | -20.21 | -24.59 to -15.84 | Yes | <0.0001 |
| Mock:cat2-1 **vs.** B.c:CAT2::CAT2N cat2-1#1 | -33.32 | -37.69 to -28.95 | Yes | <0.0001 |
| Mock:cat2-1 **vs.** B.c:CAT2::CAT2N cat2-1#2 | -34.41 | -38.78 to -30.04 | Yes | <0.0001 |
| Mock:CAT2::CAT2N cat2-1#1 **vs.** Mock:CAT2::CAT2N cat2-1#2 | 0.005133 | -4.367 to 4.377 | No | >0.9999 |
| Mock:CAT2::CAT2N cat2-1#1 **vs.** B.c:WT | -46.84 | -51.22 to -42.47 | Yes | <0.0001 |
| Mock:CAT2::CAT2N cat2-1#1 **vs.** B.c:cat2-1 | -20 | -24.37 to -15.63 | Yes | <0.0001 |
| Mock:CAT2::CAT2N cat2-1#1 **vs.** B.c:CAT2::CAT2N cat2-1#1 | -33.1 | -37.48 to -28.73 | Yes | <0.0001 |
| Mock:CAT2::CAT2N cat2-1#1 **vs.** B.c:CAT2::CAT2N cat2-1#2 | -34.19 | -38.57 to -29.82 | Yes | <0.0001 |
| Mock:CAT2::CAT2N cat2-1#2 **vs.** B.c:WT | -46.85 | -51.22 to -42.48 | Yes | <0.0001 |
| Mock:CAT2::CAT2N cat2-1#2 **vs.** B.c:cat2-1 | -20 | -24.38 to -15.63 | Yes | <0.0001 |
| Mock:CAT2::CAT2N cat2-1#2 **vs.** B.c:CAT2::CAT2N cat2-1#1 | -33.11 | -37.48 to -28.74 | Yes | <0.0001 |
| Mock:CAT2::CAT2N cat2-1#2 **vs.** B.c:CAT2::CAT2N cat2-1#2 | -34.2 | -38.57 to -29.83 | Yes | <0.0001 |
| B.c:WT **vs.** B.c:cat2-1 | 26.84 | 22.47 to 31.22 | Yes | <0.0001 |
| B.c:WT **vs.** B.c:CAT2::CAT2N cat2-1#1 | 13.74 | 9.367 to 18.11 | Yes | <0.0001 |
| B.c:WT **vs.** B.c:CAT2::CAT2N cat2-1#2 | 12.65 | 8.277 to 17.02 | Yes | <0.0001 |
| B.c:cat2-1 **vs.** B.c:CAT2::CAT2N cat2-1#1 | -13.11 | -17.48 to -8.733 | Yes | <0.0001 |
| B.c:cat2-1 **vs.** B.c:CAT2::CAT2N cat2-1#2 | -14.2 | -18.57 to -9.823 | Yes | <0.0001 |
| B.c:CAT2::CAT2N cat2-1#1 **vs.** B.c:CAT2::CAT2N cat2-1#2 | -1.089 | -5.462 to 3.283 | No | 0.9856 |

**Figure 5a**

| **Tukey's multiple comparisons test** | **Mean Diff.** | **95.00% CI of diff.** | **Significant?** | ***p* Value** |
| --- | --- | --- | --- | --- |
| Mock:WT **vs.** Mock:CAT2-N-SKL#1 | -0.2557 | -0.5458 to 0.03446 | No | 0.0966 |
| Mock:WT **vs.** Mock:CAT2-N-SKL#2 | -0.2283 | -0.5185 to 0.0618 | No | 0.1596 |
| Mock:WT **vs.** B.c:WT | -0.8293 | -1.119 to -0.5392 | Yes | <0.0001 |
| Mock:WT **vs.** B.c:CAT2-N-SKL#1 | -1.395 | -1.685 to -1.105 | Yes | <0.0001 |
| Mock:WT **vs.** B.c:CAT2-N-SKL#2 | -1.601 | -1.891 to -1.311 | Yes | <0.0001 |
| Mock:CAT2-N-SKL#1 **vs.** Mock:CAT2-N-SKL#2 | 0.02733 | -0.2628 to 0.3175 | No | 0.9995 |
| Mock:CAT2-N-SKL#1 **vs.** B.c:WT | -0.5737 | -0.8638 to -0.2835 | Yes | 0.0003 |
| Mock:CAT2-N-SKL#1 **vs.** B.c:CAT2-N-SKL#1 | -1.139 | -1.429 to -0.8489 | Yes | <0.0001 |
| Mock:CAT2-N-SKL#1 **vs.** B.c:CAT2-N-SKL#2 | -1.345 | -1.635 to -1.055 | Yes | <0.0001 |
| Mock:CAT2-N-SKL#2 **vs.** B.c:WT | -0.601 | -0.8911 to -0.3109 | Yes | 0.0002 |
| Mock:CAT2-N-SKL#2 **vs.** B.c:CAT2-N-SKL#1 | -1.166 | -1.456 to -0.8762 | Yes | <0.0001 |
| Mock:CAT2-N-SKL#2 **vs.** B.c:CAT2-N-SKL#2 | -1.372 | -1.662 to -1.082 | Yes | <0.0001 |
| B.c:WT **vs.** B.c:CAT2-N-SKL#1 | -0.5653 | -0.8555 to -0.2752 | Yes | 0.0003 |
| B.c:WT **vs.** B.c:CAT2-N-SKL#2 | -0.7713 | -1.061 to -0.4812 | Yes | <0.0001 |
| B.c:CAT2-N-SKL#1 **vs.** B.c:CAT2-N-SKL#2 | -0.206 | -0.4961 to 0.08413 | No | 0.2351 |

**Figure 5b**

| **Tukey's multiple comparisons test** | **Mean Diff.** | **95.00% CI of diff.** | **Significant?** | ***p* Value** |
| --- | --- | --- | --- | --- |
| Mock:WT **vs.** Mock:CAT2-N-SKL#1 | -17.2 | -66.82 to 32.41 | No | 0.8449 |
| Mock:WT **vs.** Mock:CAT2-N-SKL#2 | -13.43 | -63.05 to 36.18 | No | 0.9367 |
| Mock:WT **vs.** B.c:WT | -353.8 | -403.4 to -304.2 | Yes | <0.0001 |
| Mock:WT **vs.** B.c:CAT2-N-SKL#1 | -502.2 | -551.9 to -452.6 | Yes | <0.0001 |
| Mock:WT **vs.** B.c:CAT2-N-SKL#2 | -453.5 | -503.1 to -403.9 | Yes | <0.0001 |
| Mock:CAT2-N-SKL#1 **vs.** Mock:CAT2-N-SKL#2 | 3.77 | -45.85 to 53.39 | No | 0.9998 |
| Mock:CAT2-N-SKL#1 **vs.** B.c:WT | -336.6 | -386.2 to -287 | Yes | <0.0001 |
| Mock:CAT2-N-SKL#1 **vs.** B.c:CAT2-N-SKL#1 | -485 | -534.7 to -435.4 | Yes | <0.0001 |
| Mock:CAT2-N-SKL#1 **vs.** B.c:CAT2-N-SKL#2 | -436.3 | -485.9 to -386.7 | Yes | <0.0001 |
| Mock:CAT2-N-SKL#2 **vs.** B.c:WT | -340.3 | -390 to -290.7 | Yes | <0.0001 |
| Mock:CAT2-N-SKL#2 **vs.** B.c:CAT2-N-SKL#1 | -488.8 | -538.4 to -439.2 | Yes | <0.0001 |
| Mock:CAT2-N-SKL#2 **vs.** B.c:CAT2-N-SKL#2 | -440.1 | -489.7 to -390.5 | Yes | <0.0001 |
| B.c:WT **vs.** B.c:CAT2-N-SKL#1 | -148.5 | -198.1 to -98.86 | Yes | <0.0001 |
| B.c:WT **vs.** B.c:CAT2-N-SKL#2 | -99.75 | -149.4 to -50.13 | Yes | 0.0002 |
| B.c:CAT2-N-SKL#1 **vs.** B.c:CAT2-N-SKL#2 | 48.73 | -0.8866 to 98.35 | No | 0.0553 |

**Figure 5f**

| **Tukey's multiple comparisons test** | **Mean Diff.** | **95.00% CI of diff.** | **Significant?** | ***p* Value** |
| --- | --- | --- | --- | --- |
| Mock:WT **vs.** Mock:CAT2-N-SKL#1 | -1.506 | -8.611 to 5.599 | No | 0.9768 |
| Mock:WT **vs.** Mock:CAT2-N-SKL#2 | -2.869 | -9.975 to 4.236 | No | 0.7503 |
| Mock:WT **vs.** B.c:WT | -54 | -61.1 to -46.89 | Yes | <0.0001 |
| Mock:WT **vs.** B.c:CAT2-N-SKL#1 | -79.87 | -86.98 to -72.77 | Yes | <0.0001 |
| Mock:WT **vs.** B.c:CAT2-N-SKL#2 | -80.31 | -87.42 to -73.21 | Yes | <0.0001 |
| Mock:CAT2-N-SKL#1 **vs.** Mock:CAT2-N-SKL#2 | -1.363 | -8.469 to 5.742 | No | 0.9849 |
| Mock:CAT2-N-SKL#1 **vs.** B.c:WT | -52.49 | -59.6 to -45.38 | Yes | <0.0001 |
| Mock:CAT2-N-SKL#1 **vs.** B.c:CAT2-N-SKL#1 | -78.37 | -85.47 to -71.26 | Yes | <0.0001 |
| Mock:CAT2-N-SKL#1 **vs.** B.c:CAT2-N-SKL#2 | -78.81 | -85.91 to -71.7 | Yes | <0.0001 |
| Mock:CAT2-N-SKL#2 **vs.** B.c:WT | -51.13 | -58.23 to -44.02 | Yes | <0.0001 |
| Mock:CAT2-N-SKL#2 **vs.** B.c:CAT2-N-SKL#1 | -77 | -84.11 to -69.9 | Yes | <0.0001 |
| Mock:CAT2-N-SKL#2 **vs.** B.c:CAT2-N-SKL#2 | -77.44 | -84.55 to -70.34 | Yes | <0.0001 |
| B.c:WT **vs.** B.c:CAT2-N-SKL#1 | -25.88 | -32.98 to -18.77 | Yes | <0.0001 |
| B.c:WT **vs.** B.c:CAT2-N-SKL#2 | -26.32 | -33.42 to -19.21 | Yes | <0.0001 |
| B.c:CAT2-N-SKL#1 **vs.** B.c:CAT2-N-SKL#2 | -0.44 | -7.545 to 6.665 | No | >0.9999 |

**Figure 6b**

| **Tukey's multiple comparisons test** | **Mean Diff.** | **95.00% CI of diff.** | **Significant?** | ***p* Value** |
| --- | --- | --- | --- | --- |
| Bc:WT **vs.** Bc:35S::CAT2-N-SKL #1 | 0.4821 | 0.2426 to 0.7216 | Yes | <0.0001 |
| Bc:WT **vs.** Bc:35S::CAT2-N-SKL #1 | 0.4266 | 0.1871 to 0.6661 | Yes | <0.0001 |
| Bc:WT **vs.** Bc+SA:WT | -1.388 | -1.628 to -1.149 | Yes | <0.0001 |
| Bc:WT **vs.** Bc+SA:35S::CAT2-N-SKL #1 | 0.04578 | -0.1938 to 0.2853 | No | 0.9929 |
| Bc:WT **vs.** Bc+SA:35S::CAT2-N-SKL #1 | 0.0211 | -0.2184 to 0.2606 | No | 0.9998 |
| Bc:35S::CAT2-N-SKL #1 **vs.** Bc:35S::CAT2-N-SKL #1 | -0.05549 | -0.295 to 0.184 | No | 0.983 |
| Bc:35S::CAT2-N-SKL #1 **vs.** Bc+SA:WT | -1.87 | -2.11 to -1.631 | Yes | <0.0001 |
| Bc:35S::CAT2-N-SKL #1 **vs.** Bc+SA:35S::CAT2-N-SKL #1 | -0.4363 | -0.6758 to -0.1968 | Yes | <0.0001 |
| Bc:35S::CAT2-N-SKL #1 **vs.** Bc+SA:35S::CAT2-N-SKL #1 | -0.461 | -0.7005 to -0.2215 | Yes | <0.0001 |
| Bc:35S::CAT2-N-SKL #1 **vs.** Bc+SA:WT | -1.815 | -2.054 to -1.575 | Yes | <0.0001 |
| Bc:35S::CAT2-N-SKL #1 **vs.** Bc+SA:35S::CAT2-N-SKL #1 | -0.3808 | -0.6204 to -0.1413 | Yes | 0.0003 |
| Bc:35S::CAT2-N-SKL #1 **vs.** Bc+SA:35S::CAT2-N-SKL #1 | -0.4055 | -0.645 to -0.166 | Yes | <0.0001 |
| Bc+SA:WT **vs.** Bc+SA:35S::CAT2-N-SKL #1 | 1.434 | 1.194 to 1.673 | Yes | <0.0001 |
| Bc+SA:WT **vs.** Bc+SA:35S::CAT2-N-SKL #1 | 1.409 | 1.17 to 1.649 | Yes | <0.0001 |
| Bc+SA:35S::CAT2-N-SKL #1 **vs.** Bc+SA:35S::CAT2-N-SKL #1 | -0.02468 | -0.2642 to 0.2149 | No | 0.9996 |

**Figure 6c**

| **Tukey's multiple comparisons test** | **Mean Diff.** | **95.00% CI of diff.** | **Significant?** | ***p* Value** |
| --- | --- | --- | --- | --- |
| Mock:WT **vs.** Mock:CAT2-N-SKL#1 | -0.1887 | -0.4321 to 0.05474 | No | 0.2094 |
| Mock:WT **vs.** Mock:CAT2-N-SKL#2 | -0.2153 | -0.4587 to 0.02808 | No | 0.1075 |
| Mock:WT **vs.** B.c:WT | -0.8037 | -1.047 to -0.5603 | Yes | <0.0001 |
| Mock:WT **vs.** B.c:CAT2-N-SKL#1 | -1.384 | -1.628 to -1.141 | Yes | <0.0001 |
| Mock:WT **vs.** B.c:CAT2-N-SKL#2 | -1.286 | -1.529 to -1.042 | Yes | <0.0001 |
| Mock:WT **vs.** B.c+SA:WT | -0.33 | -0.5734 to -0.08659 | Yes | 0.0039 |
| Mock:WT **vs.** B.c+SA:CAT2-N-SKL#1 | -1.017 | -1.261 to -0.7739 | Yes | <0.0001 |
| Mock:WT **vs.** B.c+SA:CAT2-N-SKL#2 | -0.893 | -1.136 to -0.6496 | Yes | <0.0001 |
| Mock:CAT2-N-SKL#1 **vs.** Mock:CAT2-N-SKL#2 | -0.02667 | -0.2701 to 0.2167 | No | >0.9999 |
| Mock:CAT2-N-SKL#1 **vs.** B.c:WT | -0.615 | -0.8584 to -0.3716 | Yes | <0.0001 |
| Mock:CAT2-N-SKL#1 **vs.** B.c:CAT2-N-SKL#1 | -1.196 | -1.439 to -0.9523 | Yes | <0.0001 |
| Mock:CAT2-N-SKL#1 **vs.** B.c:CAT2-N-SKL#2 | -1.097 | -1.34 to -0.8536 | Yes | <0.0001 |
| Mock:CAT2-N-SKL#1 **vs.** B.c+SA:WT | -0.1413 | -0.3847 to 0.1021 | No | 0.5411 |
| Mock:CAT2-N-SKL#1 **vs.** B.c+SA:CAT2-N-SKL#1 | -0.8287 | -1.072 to -0.5853 | Yes | <0.0001 |
| Mock:CAT2-N-SKL#1 **vs.** B.c+SA:CAT2-N-SKL#2 | -0.7043 | -0.9477 to -0.4609 | Yes | <0.0001 |
| Mock:CAT2-N-SKL#2 **vs.** B.c:WT | -0.5883 | -0.8317 to -0.3449 | Yes | <0.0001 |
| Mock:CAT2-N-SKL#2 **vs.** B.c:CAT2-N-SKL#1 | -1.169 | -1.412 to -0.9256 | Yes | <0.0001 |
| Mock:CAT2-N-SKL#2 **vs.** B.c:CAT2-N-SKL#2 | -1.07 | -1.314 to -0.8269 | Yes | <0.0001 |
| Mock:CAT2-N-SKL#2 **vs.** B.c+SA:WT | -0.1147 | -0.3581 to 0.1287 | No | 0.7665 |
| Mock:CAT2-N-SKL#2 **vs.** B.c+SA:CAT2-N-SKL#1 | -0.802 | -1.045 to -0.5586 | Yes | <0.0001 |
| Mock:CAT2-N-SKL#2 **vs.** B.c+SA:CAT2-N-SKL#2 | -0.6777 | -0.9211 to -0.4343 | Yes | <0.0001 |
| B.c:WT **vs.** B.c:CAT2-N-SKL#1 | -0.5807 | -0.8241 to -0.3373 | Yes | <0.0001 |
| B.c:WT **vs.** B.c:CAT2-N-SKL#2 | -0.482 | -0.7254 to -0.2386 | Yes | <0.0001 |
| B.c:WT **vs.** B.c+SA:WT | 0.4737 | 0.2303 to 0.7171 | Yes | <0.0001 |
| B.c:WT **vs.** B.c+SA:CAT2-N-SKL#1 | -0.2137 | -0.4571 to 0.02974 | No | 0.1123 |
| B.c:WT **vs.** B.c+SA:CAT2-N-SKL#2 | -0.08933 | -0.3327 to 0.1541 | No | 0.9231 |
| B.c:CAT2-N-SKL#1 **vs.** B.c:CAT2-N-SKL#2 | 0.09867 | -0.1447 to 0.3421 | No | 0.8758 |
| B.c:CAT2-N-SKL#1 **vs.** B.c+SA:WT | 1.054 | 0.8109 to 1.298 | Yes | <0.0001 |
| B.c:CAT2-N-SKL#1 **vs.** B.c+SA:CAT2-N-SKL#1 | 0.367 | 0.1236 to 0.6104 | Yes | 0.0013 |
| B.c:CAT2-N-SKL#1 **vs.** B.c+SA:CAT2-N-SKL#2 | 0.4913 | 0.2479 to 0.7347 | Yes | <0.0001 |
| B.c:CAT2-N-SKL#2 **vs.** B.c+SA:WT | 0.9557 | 0.7123 to 1.199 | Yes | <0.0001 |
| B.c:CAT2-N-SKL#2 **vs.** B.c+SA:CAT2-N-SKL#1 | 0.2683 | 0.02492 to 0.5117 | Yes | 0.0245 |
| B.c:CAT2-N-SKL#2 **vs.** B.c+SA:CAT2-N-SKL#2 | 0.3927 | 0.1493 to 0.6361 | Yes | 0.0006 |
| B.c+SA:WT **vs.** B.c+SA:CAT2-N-SKL#1 | -0.6873 | -0.9307 to -0.4439 | Yes | <0.0001 |
| B.c+SA:WT **vs.** B.c+SA:CAT2-N-SKL#2 | -0.563 | -0.8064 to -0.3196 | Yes | <0.0001 |
| B.c+SA:CAT2-N-SKL#1 **vs.** B.c+SA:CAT2-N-SKL#2 | 0.1243 | -0.1191 to 0.3677 | No | 0.6879 |

**Figure 6d**

| **Tukey's multiple comparisons test** | **Mean Diff.** | **95.00% CI of diff.** | **Significant?** | ***p* Value** |
| --- | --- | --- | --- | --- |
| Mock:WT **vs.** Mock:CAT2-N-SKL#1 | -12.5 | -48.74 to 23.73 | No | 0.9441 |
| Mock:WT **vs.** Mock:CAT2-N-SKL#2 | -12.78 | -49.02 to 23.45 | No | 0.9372 |
| Mock:WT **vs.** B.c:WT | -351.2 | -387.4 to -314.9 | Yes | <0.0001 |
| Mock:WT **vs.** B.c:CAT2-N-SKL#1 | -448.4 | -484.6 to -412.1 | Yes | <0.0001 |
| Mock:WT **vs.** B.c:CAT2-N-SKL#2 | -425 | -461.2 to -388.7 | Yes | <0.0001 |
| Mock:WT **vs.** B.c+SA:WT | -132.5 | -168.7 to -96.25 | Yes | <0.0001 |
| Mock:WT **vs.** B.c+SA:CAT2-N-SKL#1 | -300.2 | -336.4 to -263.9 | Yes | <0.0001 |
| Mock:WT **vs.** B.c+SA:CAT2-N-SKL#2 | -289.3 | -325.5 to -253 | Yes | <0.0001 |
| Mock:CAT2-N-SKL#1 **vs.** Mock:CAT2-N-SKL#2 | -0.28 | -36.52 to 35.96 | No | >0.9999 |
| Mock:CAT2-N-SKL#1 **vs.** B.c:WT | -338.7 | -374.9 to -302.4 | Yes | <0.0001 |
| Mock:CAT2-N-SKL#1 **vs.** B.c:CAT2-N-SKL#1 | -435.9 | -472.1 to -399.6 | Yes | <0.0001 |
| Mock:CAT2-N-SKL#1 **vs.** B.c:CAT2-N-SKL#2 | -412.4 | -448.7 to -376.2 | Yes | <0.0001 |
| Mock:CAT2-N-SKL#1 **vs.** B.c+SA:WT | -120 | -156.2 to -83.75 | Yes | <0.0001 |
| Mock:CAT2-N-SKL#1 **vs.** B.c+SA:CAT2-N-SKL#1 | -287.7 | -323.9 to -251.4 | Yes | <0.0001 |
| Mock:CAT2-N-SKL#1 **vs.** B.c+SA:CAT2-N-SKL#2 | -276.8 | -313 to -240.5 | Yes | <0.0001 |
| Mock:CAT2-N-SKL#2 **vs.** B.c:WT | -338.4 | -374.6 to -302.2 | Yes | <0.0001 |
| Mock:CAT2-N-SKL#2 **vs.** B.c:CAT2-N-SKL#1 | -435.6 | -471.8 to -399.3 | Yes | <0.0001 |
| Mock:CAT2-N-SKL#2 **vs.** B.c:CAT2-N-SKL#2 | -412.2 | -448.4 to -375.9 | Yes | <0.0001 |
| Mock:CAT2-N-SKL#2 **vs.** B.c+SA:WT | -119.7 | -155.9 to -83.47 | Yes | <0.0001 |
| Mock:CAT2-N-SKL#2 **vs.** B.c+SA:CAT2-N-SKL#1 | -287.4 | -323.6 to -251.2 | Yes | <0.0001 |
| Mock:CAT2-N-SKL#2 **vs.** B.c+SA:CAT2-N-SKL#2 | -276.5 | -312.7 to -240.3 | Yes | <0.0001 |
| B.c:WT **vs.** B.c:CAT2-N-SKL#1 | -97.18 | -133.4 to -60.94 | Yes | <0.0001 |
| B.c:WT **vs.** B.c:CAT2-N-SKL#2 | -73.77 | -110 to -37.53 | Yes | <0.0001 |
| B.c:WT **vs.** B.c+SA:WT | 218.7 | 182.5 to 254.9 | Yes | <0.0001 |
| B.c:WT **vs.** B.c+SA:CAT2-N-SKL#1 | 51.01 | 14.77 to 87.25 | Yes | 0.0027 |
| B.c:WT **vs.** B.c+SA:CAT2-N-SKL#2 | 61.9 | 25.67 to 98.14 | Yes | 0.0003 |
| B.c:CAT2-N-SKL#1 **vs.** B.c:CAT2-N-SKL#2 | 23.41 | -12.83 to 59.65 | No | 0.4099 |
| B.c:CAT2-N-SKL#1 **vs.** B.c+SA:WT | 315.9 | 279.6 to 352.1 | Yes | <0.0001 |
| B.c:CAT2-N-SKL#1 **vs.** B.c+SA:CAT2-N-SKL#1 | 148.2 | 112 to 184.4 | Yes | <0.0001 |
| B.c:CAT2-N-SKL#1 **vs.** B.c+SA:CAT2-N-SKL#2 | 159.1 | 122.8 to 195.3 | Yes | <0.0001 |
| B.c:CAT2-N-SKL#2 **vs.** B.c+SA:WT | 292.5 | 256.2 to 328.7 | Yes | <0.0001 |
| B.c:CAT2-N-SKL#2 **vs.** B.c+SA:CAT2-N-SKL#1 | 124.8 | 88.54 to 161 | Yes | <0.0001 |
| B.c:CAT2-N-SKL#2 **vs.** B.c+SA:CAT2-N-SKL#2 | 135.7 | 99.44 to 171.9 | Yes | <0.0001 |
| B.c+SA:WT **vs.** B.c+SA:CAT2-N-SKL#1 | -167.7 | -203.9 to -131.4 | Yes | <0.0001 |
| B.c+SA:WT **vs.** B.c+SA:CAT2-N-SKL#2 | -156.8 | -193 to -120.6 | Yes | <0.0001 |
| B.c+SA:CAT2-N-SKL#1 **vs.** B.c+SA:CAT2-N-SKL#2 | 10.89 | -25.34 to 47.13 | No | 0.9741 |

**Figure 6e**

| **Tukey's multiple comparisons test** | **Mean Diff.** | **95.00% CI of diff.** | **Significant?** | ***p* Value** |
| --- | --- | --- | --- | --- |
| Mock:WT **vs.** Mock:CAT2-N-SKL#1 | -1.767 | -13.26 to 9.724 | No | 0.9997 |
| Mock:WT **vs.** Mock:CAT2-N-SKL#2 | -1.69 | -13.18 to 9.801 | No | 0.9998 |
| Mock:WT **vs.** B.c:WT | -54.38 | -65.87 to -42.89 | Yes | <0.0001 |
| Mock:WT **vs.** B.c:CAT2-N-SKL#1 | -73.98 | -85.47 to -62.49 | Yes | <0.0001 |
| Mock:WT **vs.** B.c:CAT2-N-SKL#2 | -75.49 | -86.98 to -64 | Yes | <0.0001 |
| Mock:WT **vs.** B.c+SA:WT | -21.41 | -32.9 to -9.916 | Yes | 0.0001 |
| Mock:WT **vs.** B.c+SA:CAT2-N-SKL#1 | -52.4 | -63.89 to -40.91 | Yes | <0.0001 |
| Mock:WT **vs.** B.c+SA:CAT2-N-SKL#2 | -51.5 | -62.99 to -40.01 | Yes | <0.0001 |
| Mock:CAT2-N-SKL#1 **vs.** Mock:CAT2-N-SKL#2 | 0.077 | -11.41 to 11.57 | No | >0.9999 |
| Mock:CAT2-N-SKL#1 **vs.** B.c:WT | -52.62 | -64.11 to -41.12 | Yes | <0.0001 |
| Mock:CAT2-N-SKL#1 **vs.** B.c:CAT2-N-SKL#1 | -72.22 | -83.71 to -60.73 | Yes | <0.0001 |
| Mock:CAT2-N-SKL#1 **vs.** B.c:CAT2-N-SKL#2 | -73.73 | -85.22 to -62.24 | Yes | <0.0001 |
| Mock:CAT2-N-SKL#1 **vs.** B.c+SA:WT | -19.64 | -31.13 to -8.15 | Yes | 0.0003 |
| Mock:CAT2-N-SKL#1 **vs.** B.c+SA:CAT2-N-SKL#1 | -50.64 | -62.13 to -39.15 | Yes | <0.0001 |
| Mock:CAT2-N-SKL#1 **vs.** B.c+SA:CAT2-N-SKL#2 | -49.73 | -61.22 to -38.24 | Yes | <0.0001 |
| Mock:CAT2-N-SKL#2 **vs.** B.c:WT | -52.69 | -64.18 to -41.2 | Yes | <0.0001 |
| Mock:CAT2-N-SKL#2 **vs.** B.c:CAT2-N-SKL#1 | -72.29 | -83.78 to -60.8 | Yes | <0.0001 |
| Mock:CAT2-N-SKL#2 **vs.** B.c:CAT2-N-SKL#2 | -73.8 | -85.29 to -62.31 | Yes | <0.0001 |
| Mock:CAT2-N-SKL#2 **vs.** B.c+SA:WT | -19.72 | -31.21 to -8.227 | Yes | 0.0003 |
| Mock:CAT2-N-SKL#2 **vs.** B.c+SA:CAT2-N-SKL#1 | -50.71 | -62.2 to -39.22 | Yes | <0.0001 |
| Mock:CAT2-N-SKL#2 **vs.** B.c+SA:CAT2-N-SKL#2 | -49.81 | -61.3 to -38.32 | Yes | <0.0001 |
| B.c:WT **vs.** B.c:CAT2-N-SKL#1 | -19.6 | -31.09 to -8.111 | Yes | 0.0003 |
| B.c:WT **vs.** B.c:CAT2-N-SKL#2 | -21.11 | -32.6 to -9.621 | Yes | 0.0001 |
| B.c:WT **vs.** B.c+SA:WT | 32.98 | 21.48 to 44.47 | Yes | <0.0001 |
| B.c:WT **vs.** B.c+SA:CAT2-N-SKL#1 | 1.978 | -9.512 to 13.47 | No | 0.9994 |
| B.c:WT **vs.** B.c+SA:CAT2-N-SKL#2 | 2.885 | -8.605 to 14.38 | No | 0.9914 |
| B.c:CAT2-N-SKL#1 **vs.** B.c:CAT2-N-SKL#2 | -1.51 | -13 to 9.98 | No | >0.9999 |
| B.c:CAT2-N-SKL#1 **vs.** B.c+SA:WT | 52.58 | 41.09 to 64.07 | Yes | <0.0001 |
| B.c:CAT2-N-SKL#1 **vs.** B.c+SA:CAT2-N-SKL#1 | 21.58 | 10.09 to 33.07 | Yes | <0.0001 |
| B.c:CAT2-N-SKL#1 **vs.** B.c+SA:CAT2-N-SKL#2 | 22.49 | 11 to 33.98 | Yes | <0.0001 |
| B.c:CAT2-N-SKL#2 **vs.** B.c+SA:WT | 54.09 | 42.6 to 65.58 | Yes | <0.0001 |
| B.c:CAT2-N-SKL#2 **vs.** B.c+SA:CAT2-N-SKL#1 | 23.09 | 11.6 to 34.58 | Yes | <0.0001 |
| B.c:CAT2-N-SKL#2 **vs.** B.c+SA:CAT2-N-SKL#2 | 24 | 12.51 to 35.49 | Yes | <0.0001 |
| B.c+SA:WT **vs.** B.c+SA:CAT2-N-SKL#1 | -31 | -42.49 to -19.51 | Yes | <0.0001 |
| B.c+SA:WT **vs.** B.c+SA:CAT2-N-SKL#2 | -30.09 | -41.58 to -18.6 | Yes | <0.0001 |
| B.c+SA:CAT2-N-SKL#1 **vs.** B.c+SA:CAT2-N-SKL#2 | 0.9067 | -10.58 to 12.4 | No | >0.9999 |

**Figure 6f**

| **Tukey's multiple comparisons test** | **Mean Diff.** | **95.00% CI of diff.** | **Significant?** | ***p* Value** |
| --- | --- | --- | --- | --- |
| Day 0:WT **vs.** Day 0:CAT2-N-SKL#1 | -0.09233 | -0.8646 to 0.6799 | No | 0.9983 |
| Day 0:WT **vs.** Day 0:CAT2-N-SKL#2 | -0.08367 | -0.8559 to 0.6886 | No | 0.9989 |
| Day 0:WT **vs.** Day 3:WT | -3.351 | -4.124 to -2.579 | Yes | <0.0001 |
| Day 0:WT **vs.** Day 3:CAT2-N-SKL#1 | -3.476 | -4.249 to -2.704 | Yes | <0.0001 |
| Day 0:WT **vs.** Day 3:CAT2-N-SKL#2 | -3.381 | -4.154 to -2.609 | Yes | <0.0001 |
| Day 0:CAT2-N-SKL#1 **vs.** Day 0:CAT2-N-SKL#2 | 0.008667 | -0.7636 to 0.7809 | No | >0.9999 |
| Day 0:CAT2-N-SKL#1 **vs.** Day 3:WT | -3.259 | -4.031 to -2.487 | Yes | <0.0001 |
| Day 0:CAT2-N-SKL#1 **vs.** Day 3:CAT2-N-SKL#1 | -3.384 | -4.156 to -2.612 | Yes | <0.0001 |
| Day 0:CAT2-N-SKL#1 **vs.** Day 3:CAT2-N-SKL#2 | -3.289 | -4.061 to -2.517 | Yes | <0.0001 |
| Day 0:CAT2-N-SKL#2 **vs.** Day 3:WT | -3.268 | -4.04 to -2.495 | Yes | <0.0001 |
| Day 0:CAT2-N-SKL#2 **vs.** Day 3:CAT2-N-SKL#1 | -3.393 | -4.165 to -2.62 | Yes | <0.0001 |
| Day 0:CAT2-N-SKL#2 **vs.** Day 3:CAT2-N-SKL#2 | -3.298 | -4.07 to -2.525 | Yes | <0.0001 |
| Day 3:WT **vs.** Day 3:CAT2-N-SKL#1 | -0.1251 | -0.8973 to 0.6472 | No | 0.9929 |
| Day 3:WT **vs.** Day 3:CAT2-N-SKL#2 | -0.03007 | -0.8023 to 0.7422 | No | >0.9999 |
| Day 3:CAT2-N-SKL#1 **vs.** Day 3:CAT2-N-SKL#2 | 0.095 | -0.6773 to 0.8673 | No | 0.998 |

**Figure 6g**

| **Tukey's multiple comparisons test** | **Mean Diff.** | **95.00% CI of diff.** | **Significant?** | ***p* Value** |
| --- | --- | --- | --- | --- |
| Mock:WT **vs.** Mock:CAT2-N-SKL#1 | -0.03783 | -13.89 to 13.82 | No | >0.9999 |
| Mock:WT **vs.** Mock:CAT2-N-SKL#2 | -0.039 | -13.9 to 13.82 | No | >0.9999 |
| Mock:WT **vs.** DC3000:WT | -107.6 | -121.5 to -93.79 | Yes | <0.0001 |
| Mock:WT **vs.** DC3000:CAT2-N-SKL#1 | -103.5 | -117.3 to -89.61 | Yes | <0.0001 |
| Mock:WT **vs.** DC3000:CAT2-N-SKL#2 | -108.3 | -122.1 to -94.4 | Yes | <0.0001 |
| Mock:CAT2-N-SKL#1 **vs.** Mock:CAT2-N-SKL#2 | -0.001167 | -13.86 to 13.85 | No | >0.9999 |
| Mock:CAT2-N-SKL#1 **vs.** DC3000:WT | -107.6 | -121.5 to -93.75 | Yes | <0.0001 |
| Mock:CAT2-N-SKL#1 **vs.** DC3000:CAT2-N-SKL#1 | -103.4 | -117.3 to -89.57 | Yes | <0.0001 |
| Mock:CAT2-N-SKL#1 **vs.** DC3000:CAT2-N-SKL#2 | -108.2 | -122.1 to -94.36 | Yes | <0.0001 |
| Mock:CAT2-N-SKL#2 **vs.** DC3000:WT | -107.6 | -121.5 to -93.75 | Yes | <0.0001 |
| Mock:CAT2-N-SKL#2 **vs.** DC3000:CAT2-N-SKL#1 | -103.4 | -117.3 to -89.57 | Yes | <0.0001 |
| Mock:CAT2-N-SKL#2 **vs.** DC3000:CAT2-N-SKL#2 | -108.2 | -122.1 to -94.36 | Yes | <0.0001 |
| DC3000:WT **vs.** DC3000:CAT2-N-SKL#1 | 4.173 | -9.683 to 18.03 | No | 0.9054 |
| DC3000:WT **vs.** DC3000:CAT2-N-SKL#2 | -0.616 | -14.47 to 13.24 | No | >0.9999 |
| DC3000:CAT2-N-SKL#1 **vs.** DC3000:CAT2-N-SKL#2 | -4.789 | -18.64 to 9.067 | No | 0.8466 |
