# Supplementary material for: Overexpressing the N‐terminus of CATALASE2 enhances plant jasmonic acid biosynthesis and resistance to necrotrophic pathogen Botrytis cinerea B05.10
Source: Mol Plant Pathol. 2021 Jul 10;22(10):1226–38. doi: 10.1111/mpp.13106 (PMC8435237; doi:10.1111/mpp.13106)
Supplement: Supplementary file 3 — TABLE S3 Two‐way analysis of variance in this study [file MPP-22-1226-s001.docx]

**Supplemental Table 3. Tables of the two-way ANOVA results in this study.**

**Figure 4d**

| **Source of Variation** | **Type III SS** | **DF** | **MS** | **F value** | **P value** |
| --- | --- | --- | --- | --- | --- |
| Treatment*Genotype | 0.079 | 3 | 0.026 | 3.491 | 0.0400 |
| Treatment | 3.703 | 1 | 3.703 | 487.990 | 0.0000 |
| Genotype | 0.556 | 3 | 0.185 | 24.417 | 0.0000 |
| Residual | 0.121 | 16 | 0.008 |  |  |

**Figure 4e**

| **Source of Variation** | **Type III SS** | **DF** | **MS** | **F value** | **P value** |
| --- | --- | --- | --- | --- | --- |
| Treatment*Genotype | 11886.390 | 3 | 3962.000 | 23.843 | 0.0000 |
| Treatment | 452356.437 | 1 | 452356.00 | 2722.134 | 0.0000 |
| Genotype | 13857.089 | 3 | 4619.000 | 27.796 | 0.0000 |
| Residual | 2659.834 | 16 | 166.177 |  |  |

**Figure 4f**

| **Source of Variation** | **Type III SS** | **DF** | **MS** | **F value** | **P value** |
| --- | --- | --- | --- | --- | --- |
| Treatment*Genotype | 532.215 | 3 | 177.405 | 74.156 | 0.0000 |
| Treatment | 6768.261 | 1 | 6768.261 | 2829.176 | 0.0000 |
| Genotype | 550.769 | 3 | 183.590 | 76.742 | 0.0000 |
| Residual | 38.277 | 16 | 2.392 |  |  |

**Figure 5a**

| **Source of Variation** | **Type III SS** | **DF** | **MS** | **F value** | **P value** |
| --- | --- | --- | --- | --- | --- |
| Treatment*Genotype | 0.2230 | 2 | 0.1110 | 9.9450 | 0.0028 |
| Treatment | 5.5800 | 1 | 5.5800 | 498.6050 | 0.0000 |
| Genotype | 0.8530 | 2 | 0.4260 | 38.0940 | 0.0000 |
| Residual | 0.1340 | 12 | 0.0110 |  |  |

**Figure 5b**

| **Source of Variation** | **Type III SS** | **DF** | **MS** | **F value** | **P value** |
| --- | --- | --- | --- | --- | --- |
| Treatment*Genotype | 13352.041 | 2 | 6676.020 | 20.397 | 0.0000 |
| Treatment | 817788.342 | 1 | 817788.342 | 2498.594 | 0.0000 |
| Genotype | 21507.912 | 2 | 10753.956 | 32.857 | 0.0000 |
| Residual | 3927.593 | 12 | 327.299 |  |  |

**Figure 5f**

| **Source of Variation** | **Type III SS** | **DF** | **MS** | **F value** | **P value** |
| --- | --- | --- | --- | --- | --- |
| Treatment*Genotype | 572.280 | 2 | 286.140 | 42.631 | 0.0000 |
| Treatment | 22009.279 | 1 | 22009.279 | 3279.092 | 0.0000 |
| Genotype | 802.443 | 2 | 401.221 | 59.777 | 0.0000 |
| Residual | 80.544 | 12 | 6.712 |  |  |

**Figure 6b**

| **Source of Variation** | **Type III SS** | **DF** | **MS** | **F value** | **P value** |
| --- | --- | --- | --- | --- | --- |
| Treatment*Genotype | 3.121 | 4 | 1.560 | 47.476 | 0.0000 |
| Treatment | 8.287 | 2 | 8.287 | 252.171 | 0.0000 |
| Genotype | 11.746 | 2 | 5.873 | 178.701 | 0.0000 |
| Residual | 1.775 | 54 | 0.033 |  |  |

**Figure 6c**

| **Source of Variation** | **Type III SS** | **DF** | **MS** | **F value** | **P value** |
| --- | --- | --- | --- | --- | --- |
| Treatment*Genotype | 0.2170 | 4 | 0.0540 | 7.4990 | 0.0010 |
| Treatment | 1.2500 | 2 | 0.6250 | 86.3120 | 0.0000 |
| Genotype | 4.7720 | 2 | 2.3860 | 329.6060 | 0.0000 |
| Residual | 0.1300 | 18 | 0.0070 |  |  |

**Figure 6d**

| **Source of Variation** | **Type III SS** | **DF** | **MS** | **F value** | **P value** |
| --- | --- | --- | --- | --- | --- |
| Treatment*Genotype | 22803.671 | 4 | 5700.918 | 35.536 | 0.0000 |
| Treatment | 725323.237 | 2 | 362661.918 | 2260.583 | 0.0000 |
| Genotype | 45767.454 | 2 | 22883.727 | 142.641 | 0.0000 |
| Residual | 28887.711 | 18 | 160.428 |  |  |

**Figure 6e**

| **Source of Variation** | **Type III SS** | **DF** | **MS** | **F value** | **P value** |
| --- | --- | --- | --- | --- | --- |
| Treatment*Genotype | 858.592 | 4 | 214.648 | 13.307 | 0.0000 |
| Treatment | 20392.943 | 2 | 10196.472 | 632.114 | 0.0000 |
| Genotype | 1846.621 | 2 | 923.311 | 57.239 | 0.0000 |
| Residual | 290.353 | 18 | 16.131 |  |  |

**Figure 6f**

| **Source of Variation** | **Type III SS** | **DF** | **MS** | **F value** | **P value** |
| --- | --- | --- | --- | --- | --- |
| Day*Genotype | 0.006 | 2 | 0.003 | 0.036 | 0.965 |
| Day | 50.330 | 1 | 50.330 | 634.734 | 0.0000 |
| Genotype | 0.035 | 2 | 0.018 | 0.224 | 0.803 |
| Residual | 0.952 | 12 | 0.079 |  |  |

**Figure 6g**

| **Source of Variation** | **Type III SS** | **DF** | **MS** | **F value** | **P value** |
| --- | --- | --- | --- | --- | --- |
| Day*Genotype | 20.491 | 2 | 10.245 | 0.401 | 0.678 |
| Day | 50973.212 | 1 | 50973.212 | 1996.954 | 0.000 |
| Genotype | 20.234 | 2 | 10.245 | 0.396 | 0.681 |
| Residual | 306.306 | 12 | 25.525 |  |  |
